# Cortico-Cardio-Respiratory Network Interactions during Anesthesia

**DOI:** 10.1371/journal.pone.0044634

**Published:** 2012-09-19

**Authors:** Yuri Shiogai, Mukesh Dhamala, Kumiko Oshima, Martin Hasler

**Affiliations:** 1 School of Computer and Communication Sciences, École Polytechnique Fédérale de Lausanne (EPFL), Lausanne, Switzerland; 2 Department of Physics and Astronomy, Neuroscience Institute, Center for Behavioral Neuroscience, Georgia State University, Atlanta, Georgia, United States of America; University of Maryland, United States of America

## Abstract

General anesthetics are used during medical and surgical procedures to reversibly induce a state of total unconsciousness in patients. Here, we investigate, from a dynamic network perspective, how the cortical and cardiovascular systems behave during anesthesia by applying nonparametric spectral techniques to cortical electroencephalography, electrocardiogram and respiratory signals recorded from anesthetized rats under two drugs, ketamine-xylazine (KX) and pentobarbital (PB). We find that the patterns of low-frequency cortico-cardio-respiratory network interactions may undergo significant changes in network activity strengths and in number of network links at different depths of anesthesia dependent upon anesthetics used.

## Introduction

Medical and surgical procedures most often involve the use of general anesthetics for reversibly producing total unconsciousness in patients. During such procedures, a multitude of physiological parameters including heart rate, respiration rate, movement and brain activity are observed to assess the level of anesthesia for the patient's safety. How anesthetics work and what quantitative measures are effective in assessing different levels of anesthesia have been the questions of intense research for years. The answers to these questions can potentially help design better depth-of-anesthesia monitoring systems and reduce the frequently-occurring intraoperative awareness [1]. The incidence of awareness may be as high as 1 to 2 for every 1000 patients [2], which translates to the occurrence of 20,000 to 40, 000 cases of anesthetic awareness annually in the United States according to the October 6, 2004's report of the Joint Commission. Here, to contribute to answering the questions about the effects of anesthetics and the network activity measures, we use nonparametric spectral techniques [3], [4] to look at the individual and network activity from simultaneously measured cortical electroencephalography (EEG), electrocardiogram (ECG) and respiratory signals recorded [5] from anesthetized rats under two anesthetics, ketamine-xylazine (KX) and pentobarbital (PB). This study is a reanalysis of the data published by Musizza et al. [5].

Central modulation of the cardiovascular system via descending signals from the brain has been well recognized for a long time [6]. Normal functioning of the cardiorespiratory system requires the regulation of oxygen, carbon dioxide, and pH. This regulation is achieved via central nervous system (CNS), particularly neuronal system in the brain stem in coordination with other higher cortical neuronal systems. The brainstem neurons control two active pumping systems: respiratory pump and cardiovascular pump. Carbon-dioxide sensitive receptors, which provide synaptic drive necessary for rhythm generation, also modulate respiratory patterns to protect the brain from changes in carbon-dioxide and pH. Respiratory modulation of baroreceptor and chemoreceptor reflexes affect heart rate and the cardiac vagal efferent nerve activity [7] Thus, at the systems level, respiratory and cardiovascular systems and the CNS controlling these systems can be considered to form a network, on which the dynamic interactions occur and determine the collective behavior of these systems. It was also reported that the human thalamus may gate respiratory sensation between cortex and brainstem [8]. In this regard, the characteristic changes in the activity of cortico-cardio-respiratory network may define the depth of anesthesia better than the individual activities can. However, the concept of network has yet to be realized in current clinical practice. Although different physiological parameters are measured, they are generally evaluated individually. Current depth-of-anesthesia monitoring systems use scalp EEG-based bispectral measure, whose reliability has recently been questioned [9].

In this paper, using Granger causality measures [10]–[12], we examine the network activity of directed interactions at different depths of anesthesia from the recordings of rats' cortical, cardiac and respiratory activities under two drugs, KX and PB. These anesthetics have often limited use only in laboratory settings with experimental animals. A previous study [5] based on the same experiment showed the existence of two distinct stages of anesthesia marked with an increase in 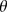-wave (3.5–7.5 Hz) as a transition from a deep stage of anesthesia to a shallow one in case of one drug KX. The phase dynamics-based approach [13], [14] was used to look at the direction of phase couplings among cortical, cardiac, and respiratory activities. In case of PB, the transition from a deep level to a shallow one was not obvious with spectral power changes. The phase-based approach was not able to unambiguously evaluate some of the directions of phase-couplings. This work characterizes all the cortico-cardio-respiratory node and network activities during different levels of anesthesia by nonparametric spectral techniques including Granger causality [3], [4].

## Materials and Methods

### Granger Causality and Nonparametric Approach

Granger causality [10] is a measure of causal or directional influence from one time series to another. Its estimation utilizes linear prediction models of measured time series. Suppose we have two dynamic processes 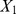 and 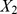 which generate the following time series: 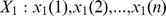 and 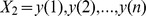. The causal influence from 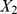 to 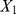 is then inferred by the reduction in the unexplained variance of the predicted 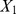 using 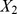 with a bivariate model (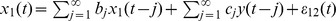) compared to the unexplained variance of the predicted 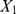 without using 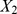 (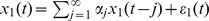). This leads to the definition of time-domain Granger causality for a pair of time series [10], [11]: 
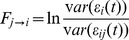
, where 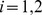 and 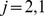. In the frequency domain, Granger causality is defined as: 
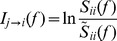
, where 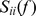 is the total power, 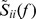 is the intrinsic power, and 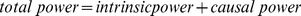 [11]. Using 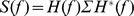, the Granger causality 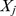 to 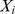 at frequency 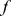 is then [11]

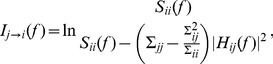
(1)where 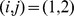 or 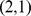, 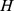 is the transfer function and 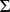 is the noise covariance function.This is pairwise or bivariate Granger causality. In a system of three or more time series, it is often desirable to find out whether a causal influence between any pair of time series is direct or mediated by others, which cannot be identified by the bivariate (or pairwise) measure of causality. Such inferences of direct or indirect can be made with conditional Granger causality [4], [12]. We can consider an example with three processes 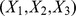, in which 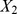 exerts a causal influence on 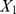 only via 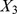. A pairwise analysis will reveal a nonzero causality from 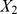 to 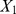 in this case. The conditional causality analysis is required to identify whether this interaction is mediated via 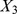. In the time domain, the Granger causality from 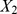 to 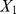 conditional on 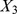 is defined as [12]: 
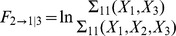
, where 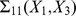 is the variance of the noise in the joint regression of 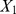 and 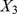, and 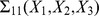 the variance in the regression of 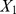, 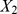, and 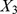, both variances being associated with 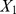 variable. In the frequency domain, the Granger causality from 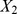 to 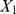 conditional on 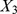 is given by [12]:
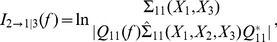
(2)where the quantities in the denominator inside the logarithm are functions of the transfer function and the noise covariance matrix. Thus, the estimation of frequency-domain Granger causality requires noise covariance (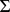) and transfer function (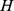), which are obtained as part of the autoregressive data-modeling. The nonparametric approach to Granger causality avoids data-modeling: data-modeling often becomes problematic [4].

The nonparametric approach is based on widely used Fourier transform for both pairwise and conditional measures [3], [4]. In this approach, spectral density matrix 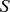 is constructed from the direct Fourier transforms: 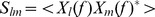, where 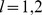, 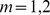, and 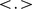 is averaging over multiple realizations. In this paper, we used windowed Fourier transforms to obtain spectral density matrix. The spectral density matrix 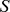 is then factored [3] into a set of unique minimum-phase functions:
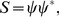
(3)where ^*^ denotes matrix adjoint, 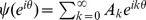 is defined on the unit circle 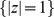, and 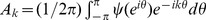 with holomorphic extension 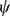 to the inner disk 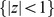 as 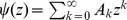 where 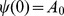, a real, upper triangular matrix with positive diagonal elements. We then obtain 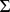 and 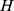 as follows [3]:
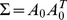
(4)

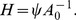
(5)The use of 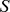, 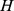, and 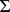 in Geweke's formulas ((1), (2)) then enables one to obtain pairwise and conditional Granger causality without explicit data-modeling. The nonparametric approach has the following advantages over the parametric approach: (i) there is no need to deal with model order calculation, and (ii) the nonparametric approach can always capture underlying complex spectral features unlike the parametric approach [3].

### Experiment

This study is a reanalysis of the data using the nonparametric spectral techniques. The details about the experiments that took place in the Institute of Pathophysiology in Ljubljana can be found in the article by Musizza and colleagues [5]. The study protocol was approved by the Panel on Ethics in biomedical research in the Institute. All the experiments [5] took place in the Institute in accordance with: State Guidelines for granting Licenses for Animal Experiments for Research Purposes, published in the Official Gazette of the Republic of Slovenia 40/85, 22/87; the Protection of Animals Act, ibid 98/99; EU regulations and recommendations, Council Directive 86/609/EEC; European Parliament resolution 2001/2259(INI); and the European Science Foundation Policy Briefing Use of Animals in Research, August 2001, Second Edition.

Simultaneous recordings of scalp EEG, ECG and respiration rate were performed on two groups of adult male Wistar rats weighing 250–300 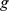 [5]. Ten rats in the first group were anesthetized with a single intraperitoneal injection of ketamine hydrochloride (45 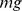 per 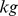 body 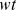) and xylazine hydrochloride (7 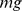 per 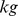 body 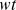)(KX group). Ten rats in the second group were anesthetized with a single intraperitoneal bolus of pentobarbital (60 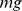 per 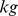 body 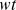) (PB group). A single time series of EEG was obtained from the recordings of a differential amplifier with one electrode over the left parietal cortex and the other over the right parietal side. The duration of recording varied from rat to rat and was more than 80 min on average both for the KX group and for the PB group.

### Data Analysis

The original time series data sampled at 1000 Hz were lowpass filtered below 45 Hz and resampled at 200 Hz. The Fourier transforms and spectral quantities (power, coherence and GC) were computed using moving time window of 1 minute with 0.5 minute overlap to look at the temporal variations. The time series within 1 min window were further broken into 4-sec segments with a 2-sec overlap. The 4-sec segments within 1 min window were used to compute the average spectral matrix. The spectral quantities such as power, coherence and Granger causality were thus derived from the average spectral matrices over time in intervals of 0.5 minute. We performed a permutation based statistical technique [4] to detect significant coherence and GC values above the background activity. For this, we considered a 10-minute segment of time series from all rats around the transition time from a deep to shallow stage, which was independently detected [5]. We put all these segments in one pool and randomized them. From this pool, we then randomly picked up segments in 1-min running windows and computed the coherence and GC for the whole 10 minutes. We then picked up maximum values of coherence and GC. We repeated all these steps for 1000 permutations and fitted the 1000 maximum values by a gamma function. From a gamma-function fit, we determined the significance threshold at 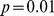. In order to find out whether the causal influence between any pair of time series which was detected above the significance threshold, is direct or mediated by others, we computed conditional GC [4].

## Results

**Figure 1:**
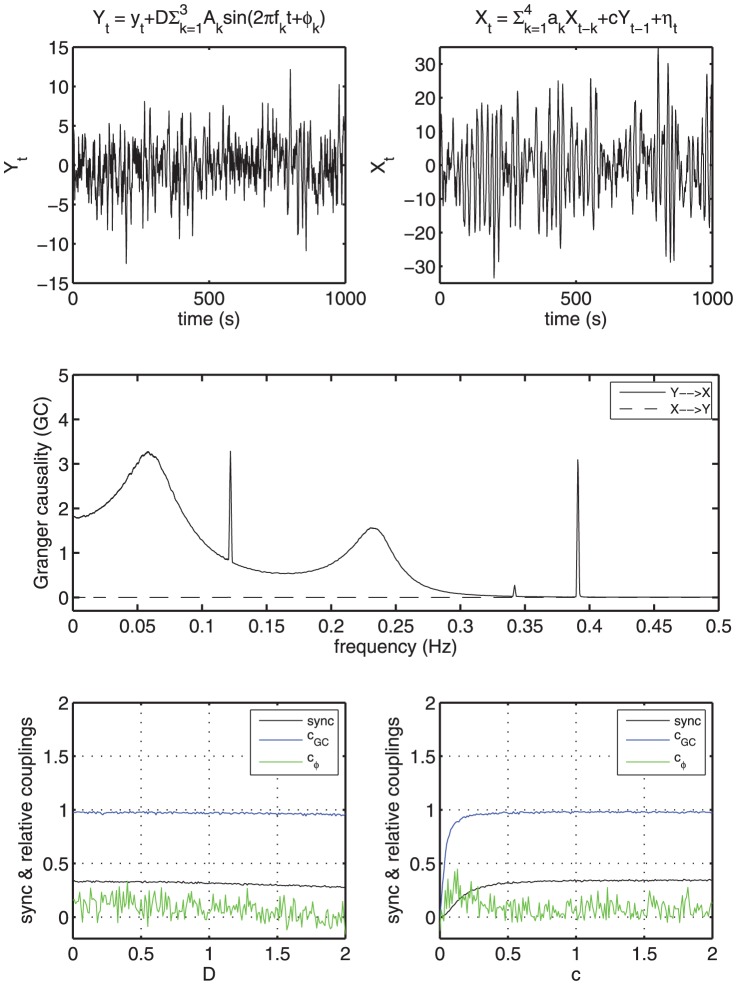
Directional measures computed from simulated stochastic and deterministic processes. The top panel shows sample time series for driving 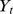 and driven 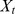 variables. As 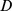 is changed from 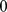, the coupled system goes from being purely stochastic to stochastic plus deterministic. The middle pannel shows that the nonparametric Granger causality correctly captures the underlying oscillatory driving direction from 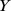 to 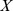 at 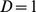 and the coupling strength, 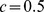. Granger causality from 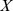 to 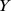 remains close to zero. The bottom left pannel shows a degree of phase synchrony (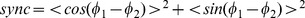) between two processes (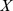 and 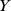) and relative couplings (directionality measures) derived from time-domain Granger causality (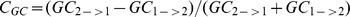) and from the phase-based technique (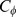). Here, 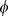
's are individual phases and 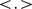 is an average. A positive value of relative couplings (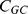, 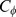) means that the coupling from 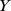 to 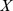 is greater than the other way around. Here, we compute sync index, 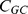 and 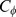 at different 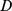, and find that GC can unabmiguously determine the directions of coupling from a coupled stochastic and deterministic system, and the phase-based technique may often fail to do so. On the right, we change the strength of coupling at 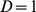 and find that the phase-based technique often fails to determine the correct directions when the coupling becomes stronger.

**Figure 2:**
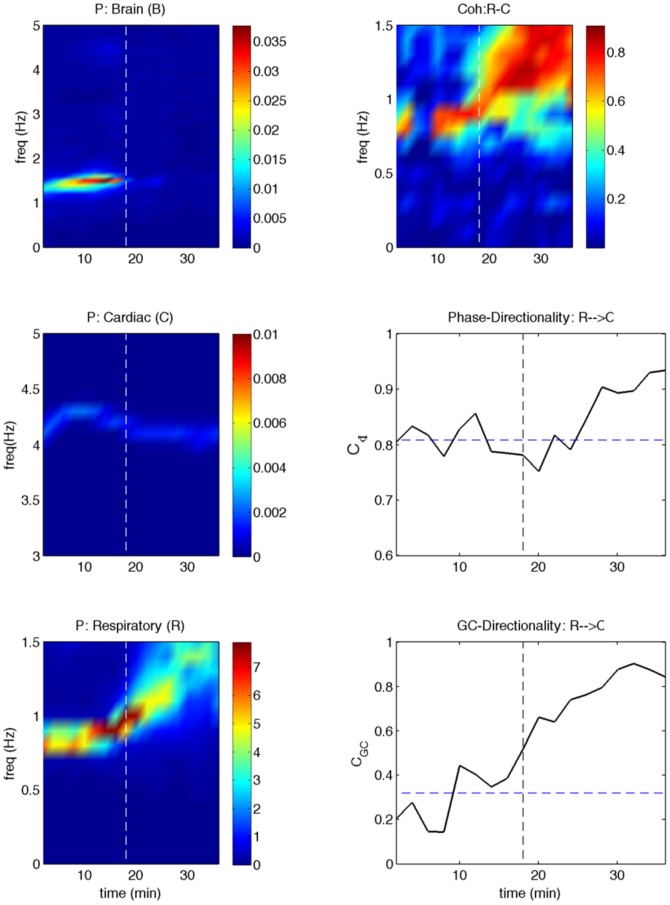
Directional measures computed from sample experimental data. Power, coherence, phase-based directionality, and GC-based directionality calculated from the data of one rat in the KX group is shown here. Time evolutions of power spectra of brain (B), cardiac (C) and respiratory (R) are shown in the first column of plots. Time evolutions of coherence spectra, phase-directionality and GC-based directionality are shown on the right column of plots. Vertical dashed lines represent the transition time from a deep to light level of anesthesia as assessed by several parameters as reported in [5]. The dashed horizontal lines in the directionality plots represent the averages over the deep stage of anesthesia. Two sample t-tests showed that the directionality averages before and after the transition over equal time-intervals are significantly different (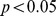). GC-based directionality around the respiration frequency (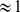 Hz) rises above 3 standard deviations at around the transition time, whereas the phase-based directionality does so at a later time.

**Figure 3:**
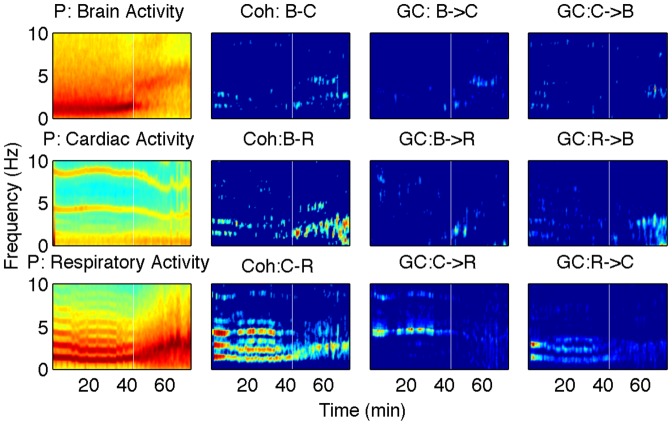
Representative spectra from the KX group. The first column power, the second column coherence, the third and forth columns pairwise GC, where C, R and B represent the cardiac activity, the respiration and the brain, respectively. The white vertical lines represent the transition from a deep to light level of anesthesia as assessed by several parameters as reported in [5].

**Figure 4:**
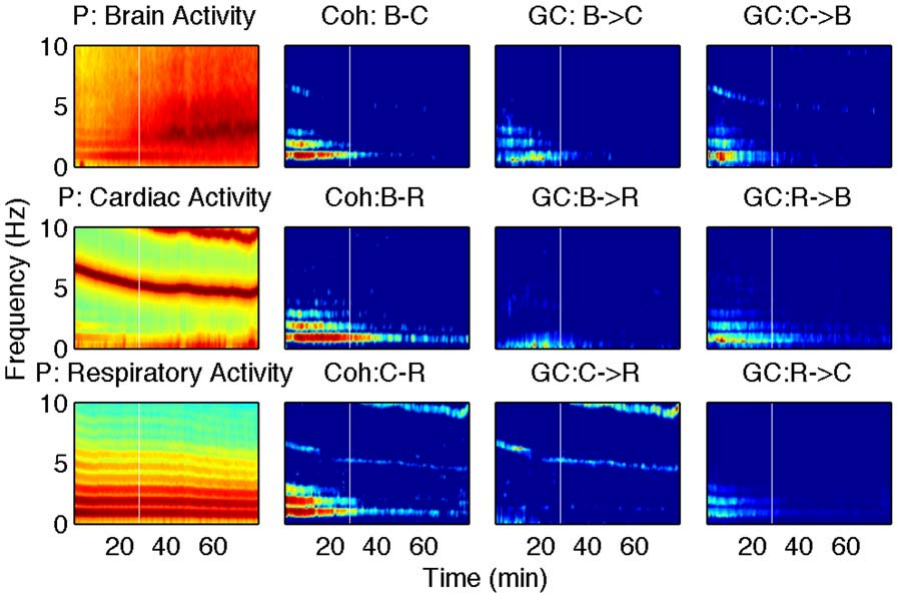
Representative spectra from the PB group. The first column shows power, the second column coherence, the third and forth columns pairwise GC.

**Figure 5:**
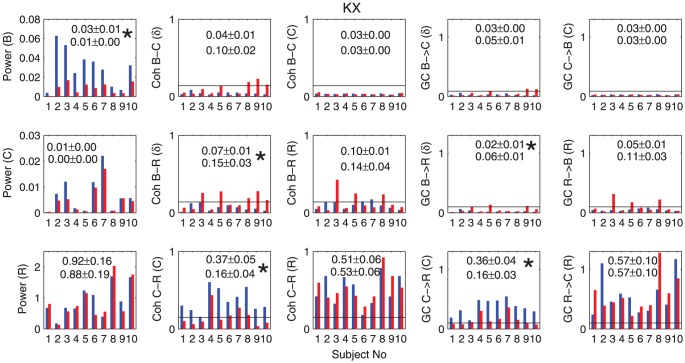
Power, coherence and GC for the KX group. The summary histograms of average power, coherence and GC before and after transition time for 10 rats in the KX group are shown here. The x-axis is the number of rats and y-axis is a value corresponding to the y-label. The symbol ‘
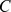
’ in y-labels means cardiac, ‘
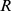
’ respiratory and ‘
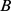
’ brain, respectively. Symbols in parenthesis represent the frequency at which average was calculated. For example, 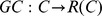 means time average of 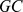 was calculated along cardiac frequency (around 4 Hz). B here means the 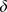-frequency range. Blue bars represent time average before the transition (deep stage) and red bars represent time average after transition (shallow stage). Horizontal black lines are threshold calculated by permutation test. Numbers in histograms are the average of time averages across all the rats and its mean errors (deep stage upper and shallow stage lower). The mark * is put beside them if the distribution of time average is significantly different (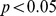) between deep and shallow stages.

**Figure 6:**
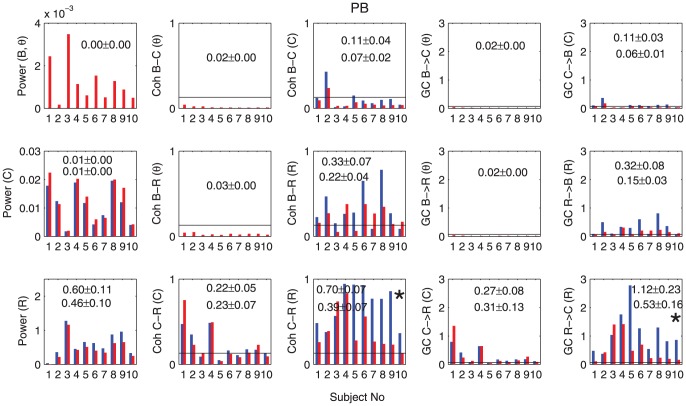
Power, coherence and GC for the PB group. The summary histograms of average power, coherence and GC before and after transition time for 10 rats the PB group are shown here. The same description of histograms as one for the KX group. Please note that that we do not have the time averages before transition (deep stage) in Power (B) since the 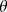-oscillations are present only in the shallow stage.

**Figure 7:**
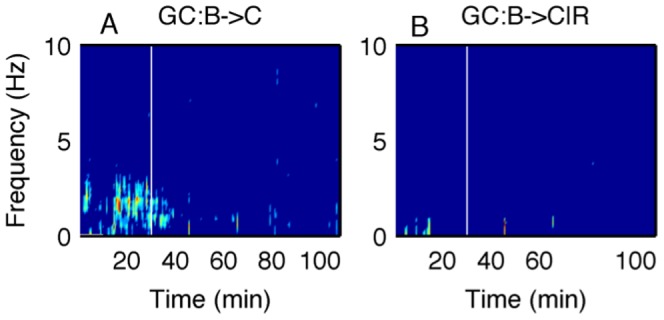
Bivariate and conditional Granger causality. A: Pairwise GC from the brain to the cardiac activity and B: GC from the brain to the cardiac activity conditional on the respiration for one rat from the KX group. The significant causality in A disappears in B, which means the causality from the brain to the cardiac activity is likely to be mediated by respiration during the deep stage of anesthesia.

Here, we first evaluate the performance of the nonparametric Granger causality [3], [4] and the phase-based approach [13] in determining directional influences from time series. We apply these techniques to synthetic data and experimental data (simultaneoulsy measured EEG-ECG-respiration data from one rat from the KX-group). Figure 1 shows such a comparison of the applications of these techniques to the time series generated by a model of a coupled system with deterministic and stochastic processes (this model is similar to the one used in [4]). The driving system is described by 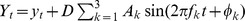 and the driven system by 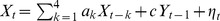, where 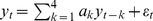. Here, 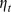 and 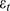 are Gaussian noise processes. At 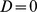, both driving and driven variables become purely stochastic and at 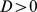, these systems show mixed behaviors (deterministic and stochastic). 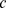 is the coupling strength. The other parameter values used here are: 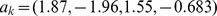, 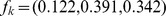, 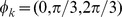, and 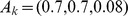. We apply the nonparametric GC and the phase-based measure to these time series at different 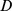
's and 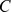
's and find out that GC is reliable whereas the phase-based technique as applied here to wide-band signals often fails to determine the directions of coupling correctly. We compare the GC-based directionality measure (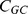) with the phase-based measure by applying to one rat's data from the KX group. The results are shown in Figure 2. This is the case where there is a very good agreement between independently assessed transition time (marked by dashed vertical lines) and the time at which the oscillatory power changes significantly in the cortex in 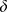-frequency range, in the heart at cardiac frequency (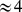 Hz), and for the respiration at respiratory frequency (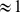 Hz) (left column of plots in Figure 2). As expected, coherence between respiratory and cardiac activities at respiratory frequency (at the top right column of Figure 2) changes significantly in going from deep to light stages. There are also significant (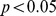) differences in directionality values between the deep and light anesthetic stages as assessed by both phase-based and nonparametric Granger causality (shown on the right in lower two panels). But, the Granger causality based measure detects the transition time better. We now present the results obtained by using these nonparametric spectral techniques. Figure 3 shows representative power, coherence and GC from the KX group. In the power plots, the spectral peaks occur around 1 Hz for the respiration and 4 Hz for the cardiac activity with the weaker higher frequency harmonics. For the brain, 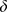 wave (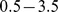 Hz) was observed during the first (deep) stage and it was weakened as the appearance of 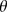 wave (3.5–5.0 Hz) at the second (shallow) stage as reported in [5]. In order to see changes in the GC around the transition time, we calculated time averaged GC before and after the transition time. At first, we detected the time varying frequency of cardiac activity (around 4 Hz), respiration (around 1 Hz) and 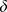 wave and 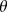 wave. The 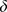 wave could be detected both at the deep stage and for 10 minutes at the shallow stage, whereas the 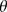 wave could be detected only at the shallow stage. At each moment, we picked up the GC which corresponded to the frequency detected above, and time-averaged separately before and after transition. The same procedure was used for the PB group and the representative plots are shown in Figure 4. We conducted Wilcoxon rank sum tests to find out differences between the time averaged values before and after the transition time. For the KX group, the GC from cardiac activity to respiration (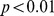) and the coherence corresponding to the cardiac frequency (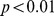) decreases significantly after the transition time, whereas there is no significant difference in the cardiac activity power. These results indicate that the coupling from cardiac to respiration becomes weaker at the shallow stage of anesthesia. There is a significant increase in the GC from the brain (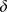 wave) to the respiration (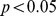) at the deep-shallow transition, whereas the power of 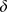 wave decreases significantly (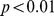). This suggests that the coupling from 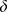 wave to respiration is strengthened at the shallow stage of anesthesia. For the PB group, the representative spectra are shown in Figure 4. The GC from respiration to cardiac activity (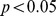) and the coherence corresponding to the respiration frequency (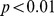) decrease significantly after the transition time, whereas there is no significant difference in the respiratory power. The average power, coherence and GC at brain frequency (B, 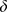, or 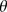), at cardiac frequency (C) and at respiratory frequency (R) are shown in Figure 3 for the KX group and in Figure 4 for the PB group. The summary histograms of average power, coherence and GC before and after transition time are shown in Figure 5 and Figure 6 for the two groups KX and PB respectively. These results indicate that the coupling from respiration to cardiac activity weakens at the shallow stage of anesthesia. It should be noted that there are 3 rats in the KX group with significant time average of Coh: B–C (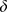) and pairwise GC: B
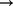
C(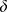) in the deep stage whereas no rat shows significance in the shallow stage. In order to check whether the influence is direct or indirect, we performed the conditional GC analyses [4]. With these analyses we found that the causal influences of the brain to the cardiac activity is mediated by respiration during the deep stage. Thus, in this stage, the brain has influence on cardiac activity via respiration only. These results on conditional causality are shown in Figure 7.

### Conclusions

**Figure 8:**
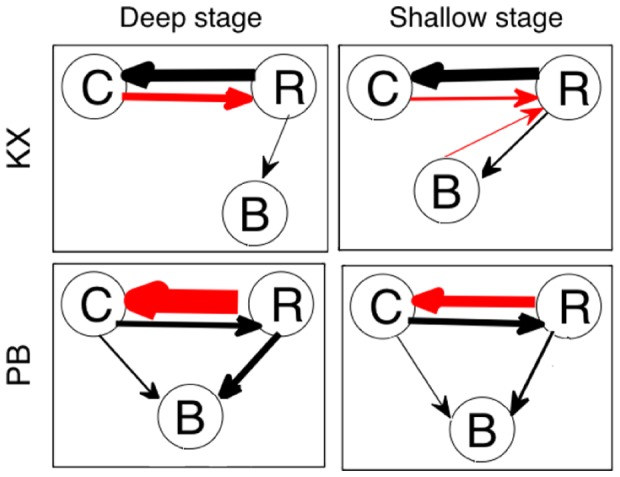
Summary of causal influences in the deep and light stages of anesthesia for both groups. An arrow is marked if at least one subject has significant time-averaged GC above threshold. The width of arrows is proportional to the magnitude of average of time-averaged GC through all the rats, which are written in Figure 5 and 6. Arrows in red color means that the magnitude of GC changes significantly between the two different stages of anesthesia.

Stages of anesthetic awareness could be assessed more reliably by bi-or multi-variate measures such as coherence and Granger causality among cortico-, cardio- and respiratory activities than the univariate measure such as power of brain, cardiac or respiratory oscillations. Please see Figures (6 and 7) for summary results and Figure S1, S2, S3, S4, S5, S6, S7, S8, S9, S10, S11, S12, S13, S14, S15, S16, S17, S18, S19, S20 in the Electronic Supplementary Material S1 0 for the results from all individual rats. Figure 8 summarizes the results from the pairwise and conditional Granger causality analyses. For the KX group, there are significant differences of GC from cardiac activity to respiration and from the brain to respiration between the two stages. After the deep-shallow transition, there is an increase in the causality from the brain (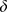) to respiration. The GC from cardiac activity to respiration decreases significantly. For the PB group, there are significant causal influences from cardiac activity to respiration, from respiration to cardiac activity, and from respiration to brain in the deep stage. After the transition, the GC from respiration to cardiac activity decreases significantly. These results can lead to the following general conclusions: (i) network interactions, especially directional influences quantified by nonparametric Granger causality, can distinguish a deep stage of anesthesia from shallow one, (ii) changes in cardiac and respiratory interactions consistently mark the transition between these two stages, and (iii) the overall cortical-cardio-respiratory network activity (number of directed links and/or strengths) may increase in going from the deep to the shallow levels with some differences in the network activity for different anesthetics. These findings not only help us understand how the cortical and cardiovascular systems behave as a network during anesthesia, but also suggest that the network activity measures might be useful for effective physiological monitoring.

## Discussion

The respiratory modulation of cardiac activity rate variability is well known as respiratory sinus arrhythmia (RSA) [15]. Cardiac influence on ventilatory dynamics has been observed and cardioventilatory coupling (CVC) has been suggested as a regular mechanism [16]. In this study, we also observed the existence of significant bidirectional influence between ECG and respiration signals for all rats. The most significant change in GC at the deep-shallow transition is the decrease of coupling from cardiac activity to respiration for the KX group and from respiration to cardiac activity for the PB group. It was shown in anaesthetized rats that a longer duration of phase synchronization between cardiac activity and respiration was observed with the concomitant decrease in respiratory frequency [17]. Our results are consistent with these previous findings. It was observed for some rats in the KX group that the coupling from 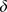 wave to respiration increases after the deep-shallow transition. Anesthetics are known to affect the chemical synapses of the neuronal systems in the brain. It is proposed that a neuronal hyperpolarization block at the level of the thalamus, or thalamocortical and corticocortical reverberant loops, could contribute to anesthetic-induced unconsciousness. This is consistent with our result of the brain weakening its influence on respiration during a deep stage of anesthesia. The hypothalamus that controls sleep/wake states is known to be a key target of anesthetics that act at GABA 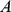 receptors [18], [19]. It is known that pentobarbital acts at receptors [18], whereas ketamine affects GABA and NMDA receptors and reduces the pre-synaptic release of glutamine [20]. The effects on respiratory and cardiovascular system are different between PB and KX. For example, the level of respiratory depression caused while anesthetized depends on which drug is used [21]. Our results of different network activity patterns for the two different anesthetics also indicate that these agents act differently, possibly at chemical synapses. There are some differences in directed interactions using the variance (amplitude)-based Granger causality and the phase-based approach as in [5]. For example, rats which have significant GC H
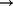
R or R
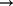
H did not have significant phase causality.The phase-based approach for directionality (or coupling) as used in Musizza et. al. [5] was not able to determine interactions with the brain in the shallow anesthetic state of the KX group and in both states (deep and shallow) of the PB group. This may indicate that the mechanism of phase coupling is different from that of amplitude coupling so that the amplitude is always affected but the phase is not. In monkey and human studies of cortical oscillation, different types of interaction (phase coupling, phase continuity and amplitude coupling) were observed depending on the distance of the cites [22]. For both KX and PB, theta waves appears not during the deep stage of anesthesia but during the light stage of anesthesia. The 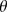 wave in the hippocampus is believed to be critical for temporal coding and decoding of active neuronal ensembles and modification of synaptic weights associated with memory processes. Various anesthetic drugs are known to affect the 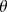 wave [23]. The appearance of 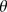 activity at the transition from a deep to shallow stage of anesthesia in the EEG signals which we analyzed may be also related with these memory processes. These results may reveal the origins of low-frequency oscillations which are commonly reported in human EEG or blood oxygen level-dependent (BOLD) fluctuating signals. For example, it was reported in [24] that slow oscillation (0.02–0.2 Hz) exists in the human cortex in the EEG during sleep. These slow oscillations in the EEG (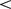
0.1 Hz) could be of cardiovascular origins because there are interactions between cardiovascular system and the brain. There is also a debate about the origins of human BOLD signal fluctuations. It was discovered that the slow fluctuations (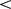
0.1 Hz) are not just random noise but the fluctuations measured in the left somatomotor cortex are specially correlated with those in the right somatomotor cortex and with medial motor areas in the absence of overt motor behavior [25]. In humans, the main cardiac (around 1 Hz) and respiratory (around 0.3 Hz) peaks were observed in the signals [26]. In this study also, we found that the slow frequency oscillations exist in the cortico-cardio-respiratory network activity. The nonparametric spectral approach including GC can be used reliably to look at the cortico-cardio-respiratory node and network activities associated with different depths of anesthetic awareness.

## Supporting Information

Electronic Supplementary Material S1 **In this Electronic Supplementary Material S1, we include the power, coherence and Granger causality results for all rats in the KX group in Fig. (S1, S2, S3, S4, S5, S6, S7, S8, S9, S10) and for rats in the PB group in Fig. (S11, S12, S13, S14, S15, S16, S17, S18, S19, S20).**
(PDF)
