# Supplementary material for: Cortico-Cardio-Respiratory Network Interactions during Anesthesia
Source: PLoS One. 2012 Sep 19;7(9):e44634. doi: 10.1371/journal.pone.0044634 (PMC3446920; doi:10.1371/journal.pone.0044634)
Supplement: Electronic Supplementary Material S1 — In this Electronic Supplementary Material S1, we include the power, coherence and Granger causality results for all rats in the KX group in Fig. (S1, S2, S3, S4, S5, S6, S7, S8, S9, S10) and for rats in the PB group in Fig. (S11, S12, S13, S14, S15, S16, S17, S18, S19, S20). (PDF) [file pone.0044634.s001.pdf]

Electronic Supplementary Material S1

Cortico-Cardio-Respiratory Network Interactions during Anesthesia

Yuri Shiogai[1], Mukesh Dhamala[2,*], Kumiko Oshima[1], Martin Hasler[1]

1. Laboratory of Nonlinear Systems (ICLANOS), ´Ecole Polytechnique F´ed´erale de Lausanne (EPFL), IC School of Computer and Communication Sciences, Lausanne, Switzerland.
2. Department of Physics and Astronomy, Neuroscience Institute, Center for Behavioral Neuroscience, Georgia State University, Atlanta, GA 30303, USA.

∗ E-mail: Corresponding mdhamala@gsu.edu

Summary

The figures below show power (P), coherence (Coh) and Granger causality (GC) spectra during deep and shallow stages of anesthesia for all twenty rats in (i) KX (Fig (S1-S10)) and (ii) PB (Fig (S11 –S20)) groups. The vertical lines represent transition times between deep and shallow stages during anesthesia. Independent tests were used to estimate these transition times. Here, B, R and H represent brain activity, respiratory activity and cardiac activity respectively.

(i) KX group (Fig S1- S10):

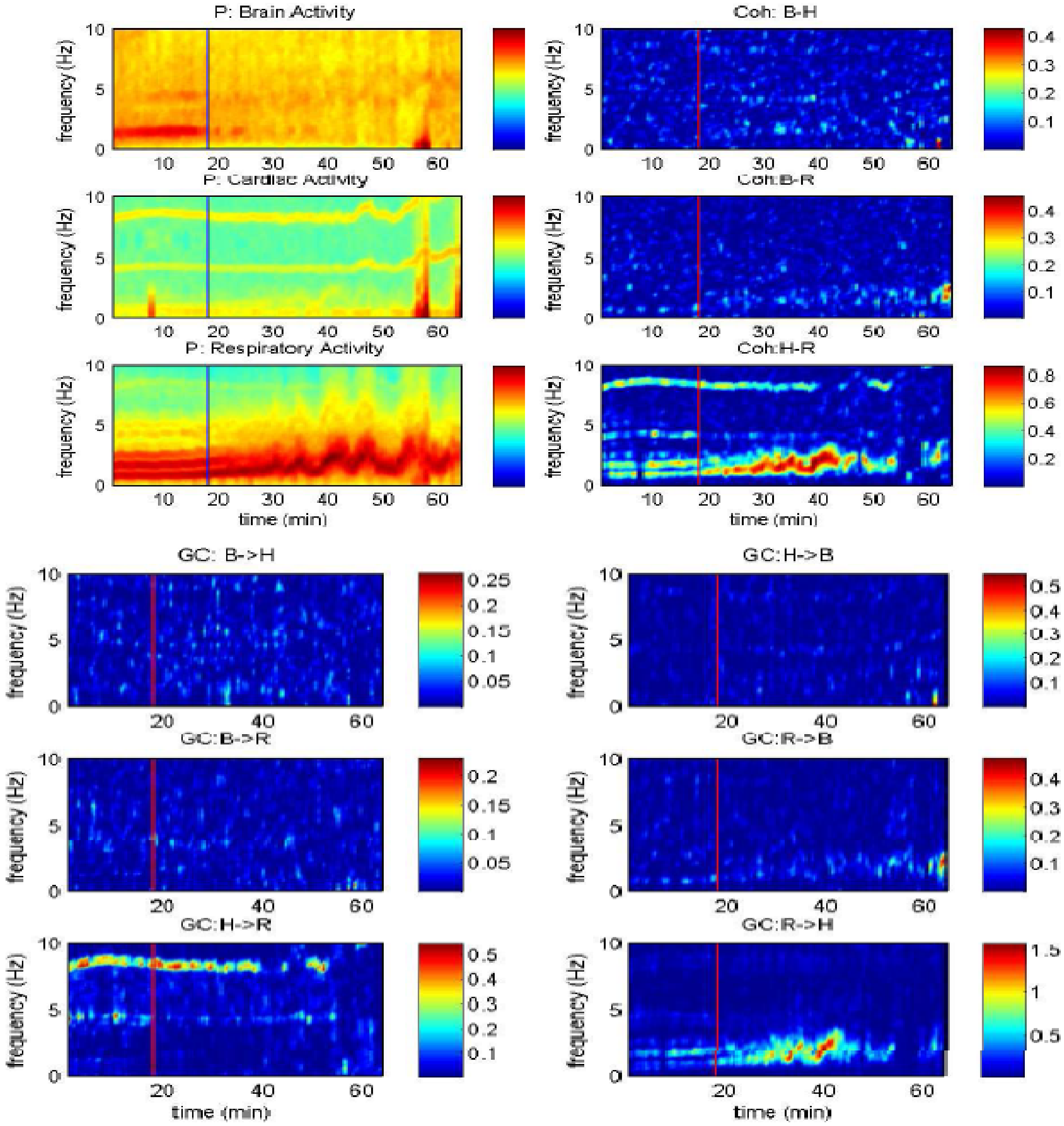

Fig S1. Rat 1 from KX group

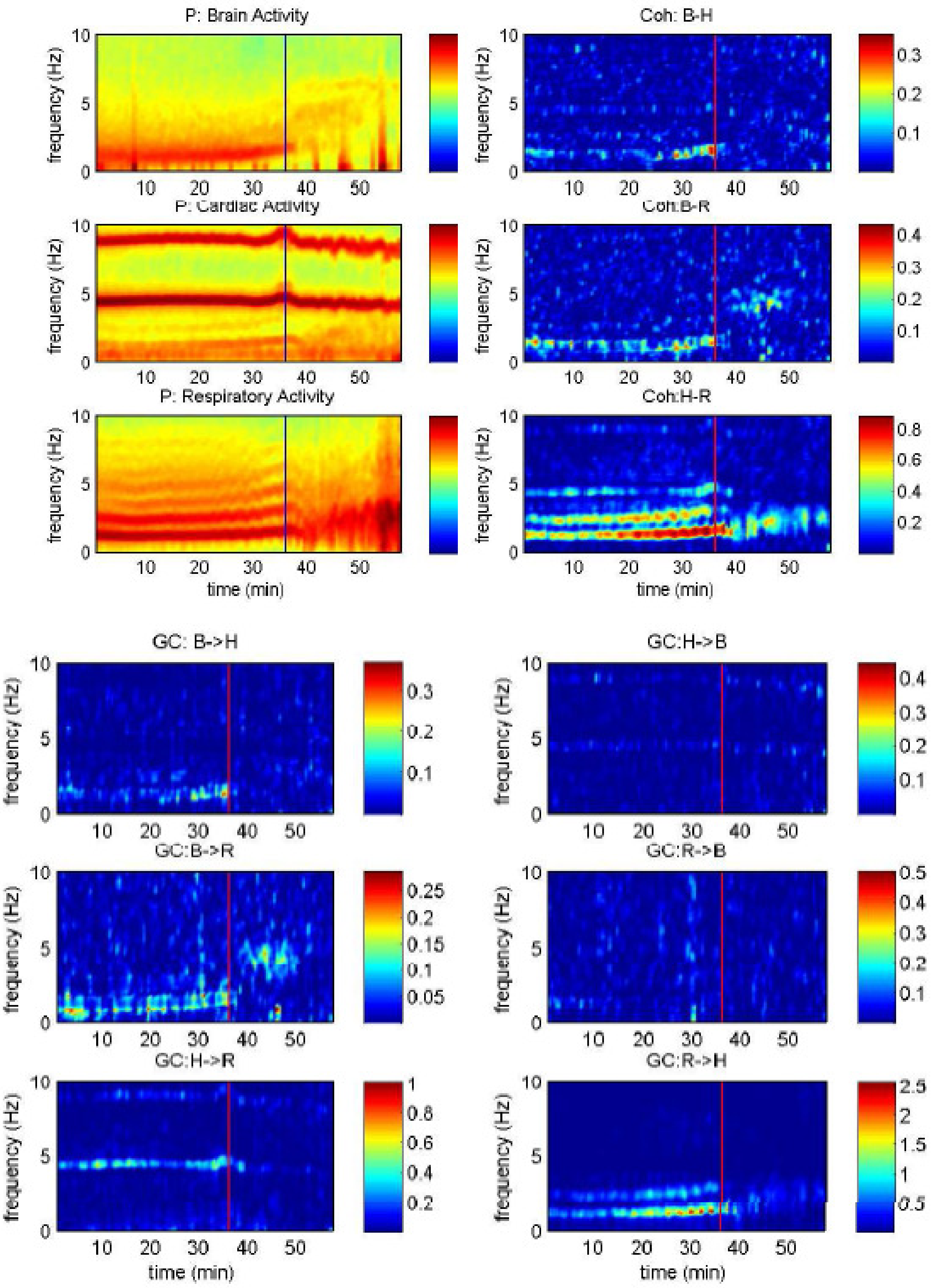

Fig S2. Rat 2 from KX group

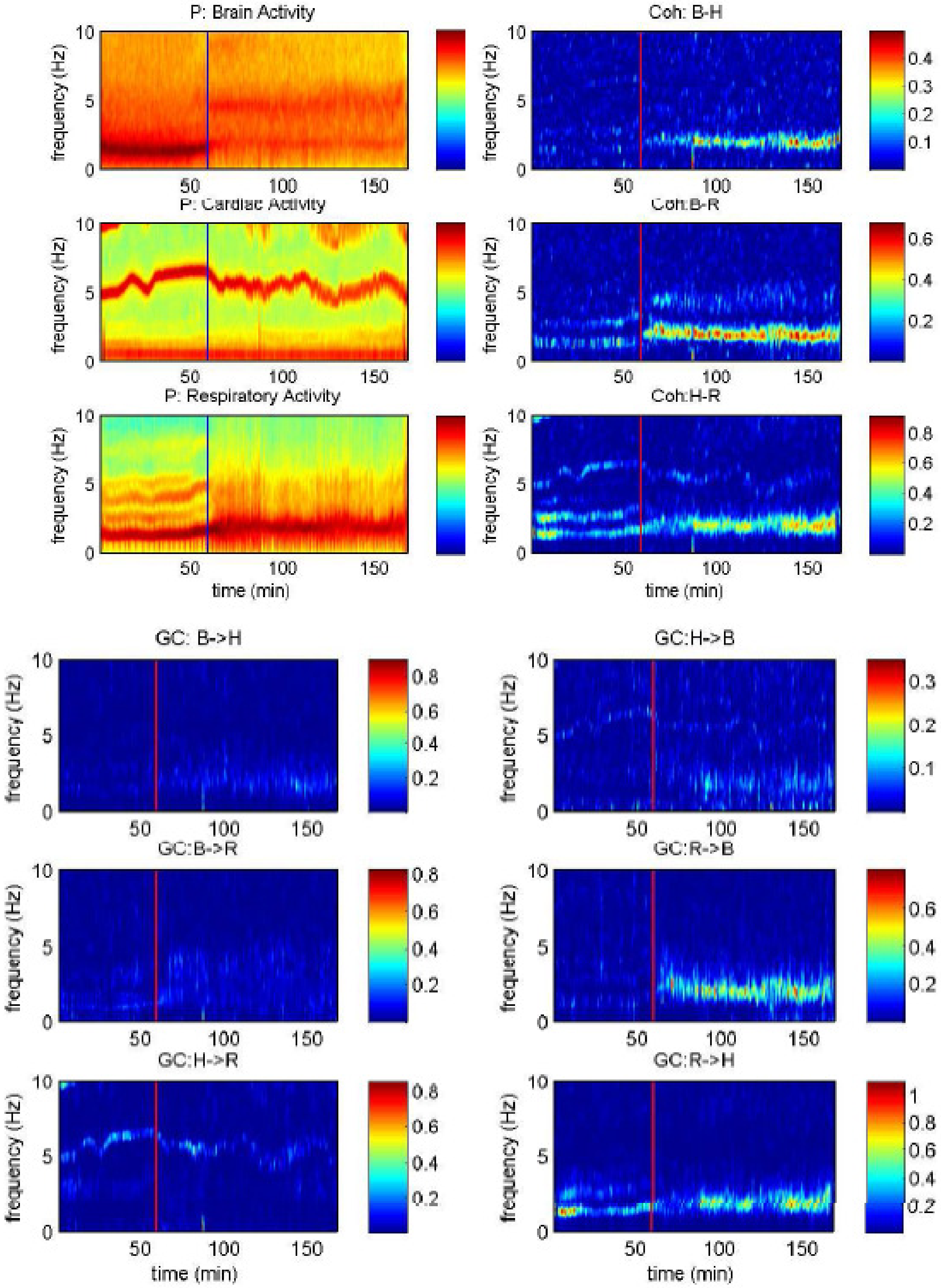

Fig S3. Rat 3 from KX group

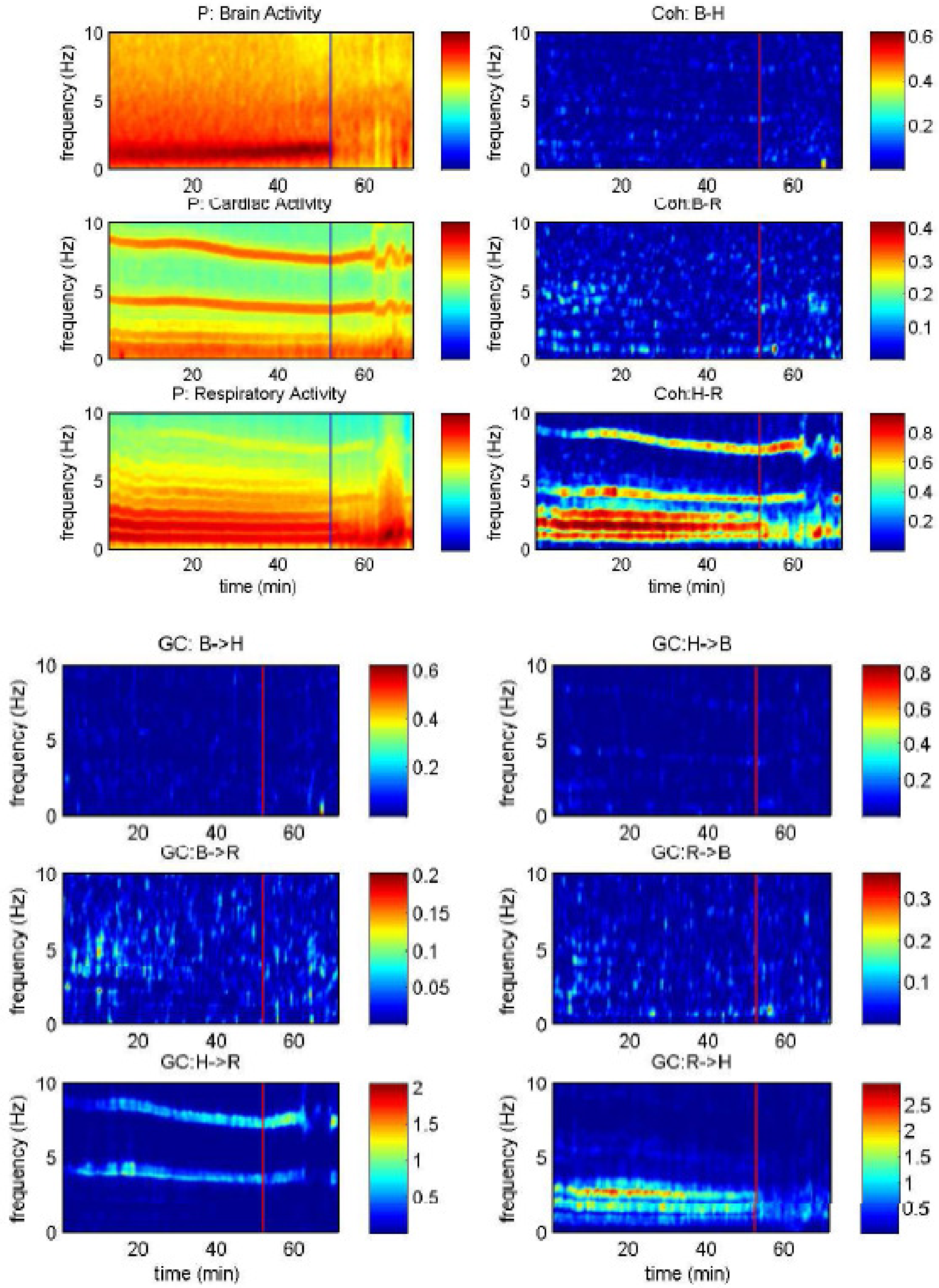

Fig S4. Rat 4 from KX group

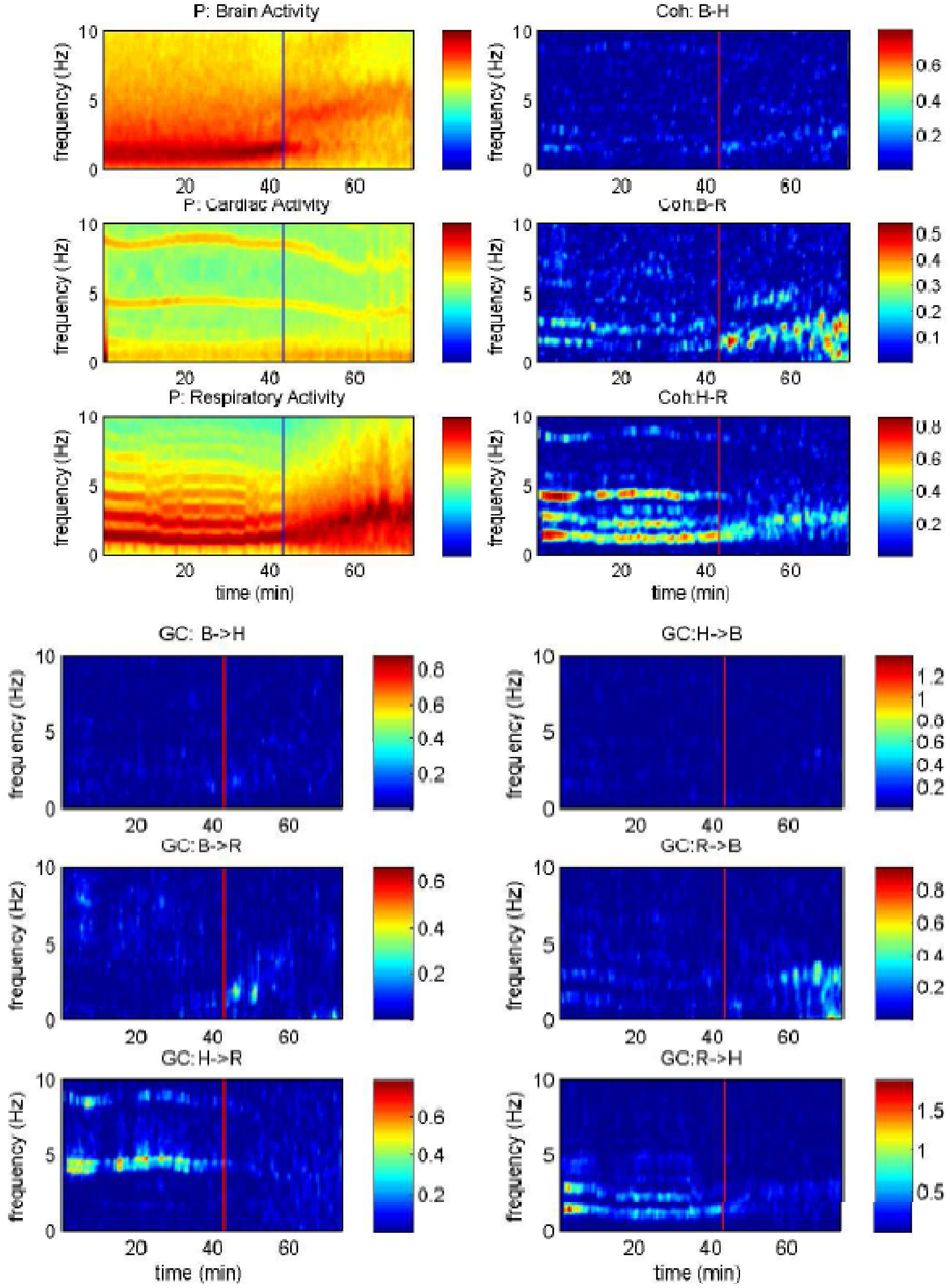

Fig S5. Rat 5 from KX group

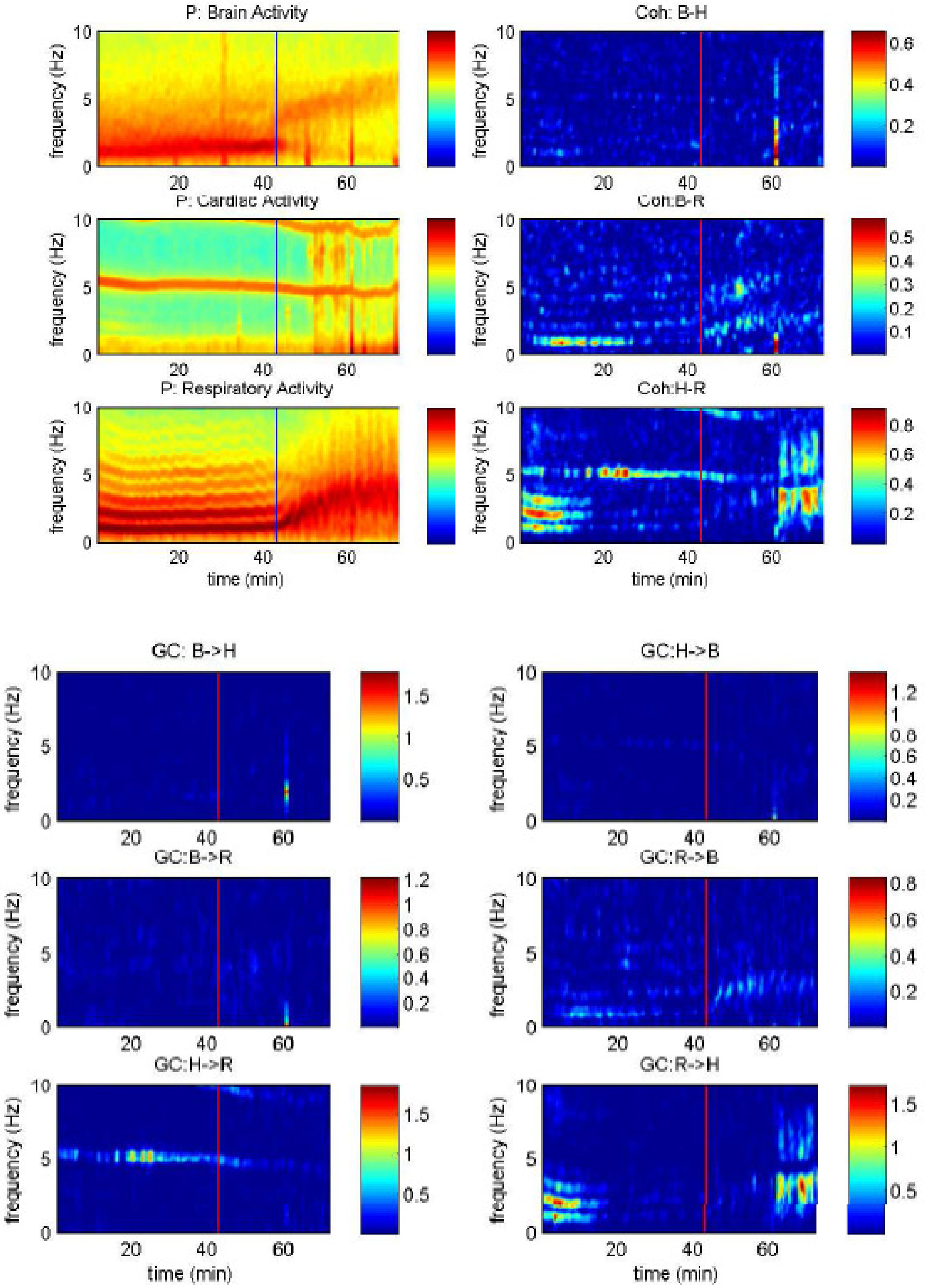

Fig S6. Rat 6 from KX group

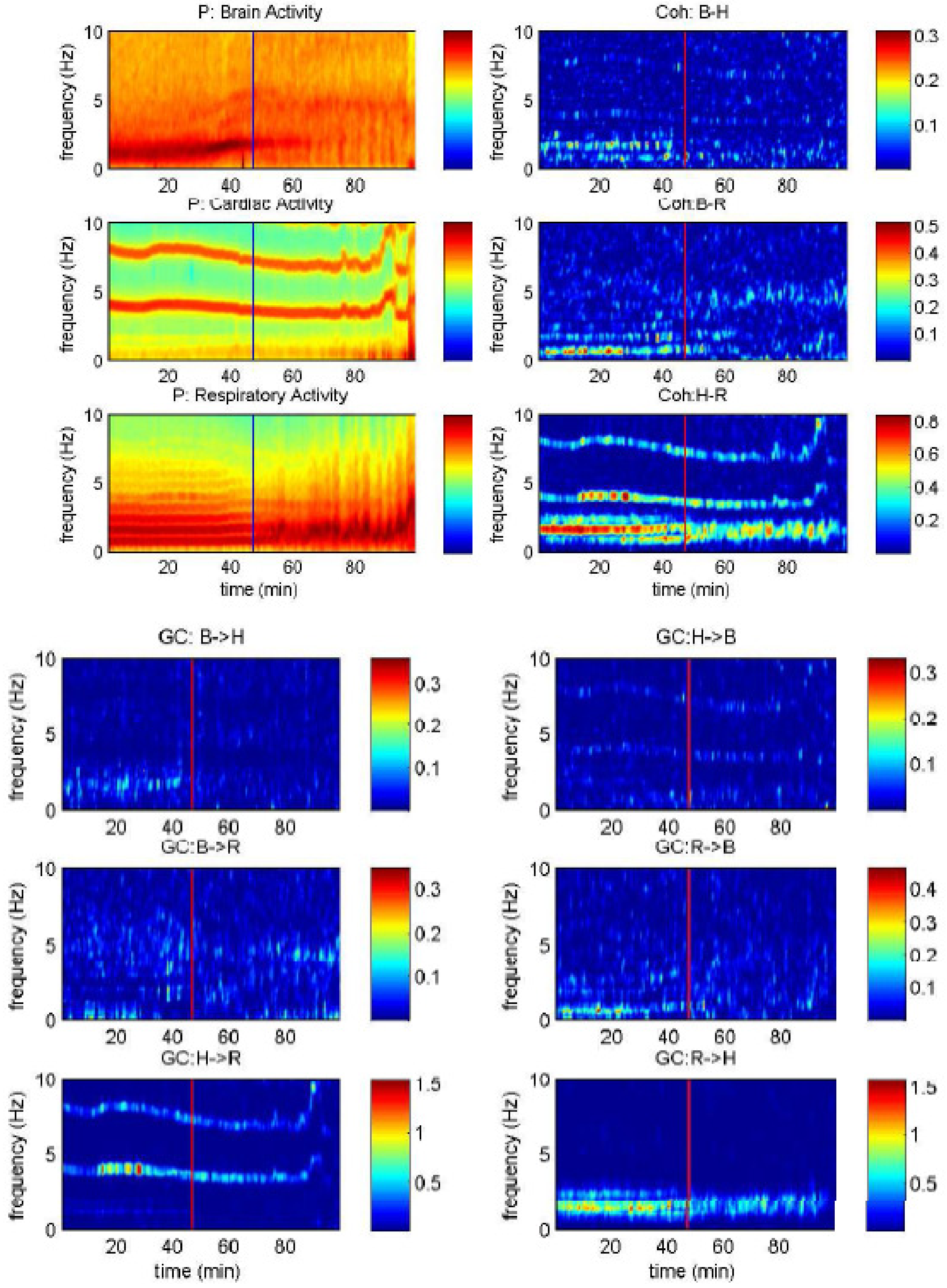

Fig S7. Rat 7 from KX group

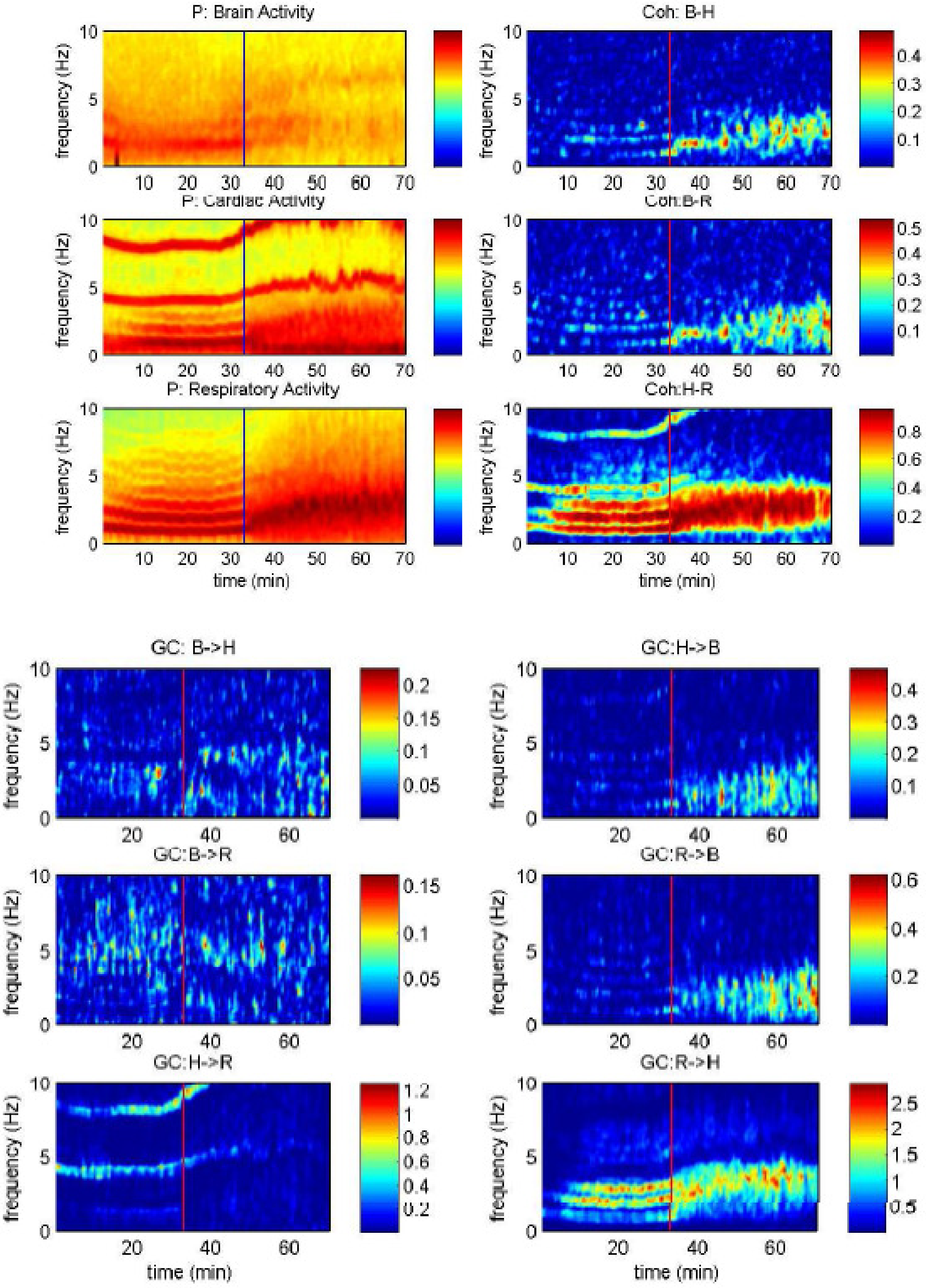

Fig S8. Rat 8 from KX group

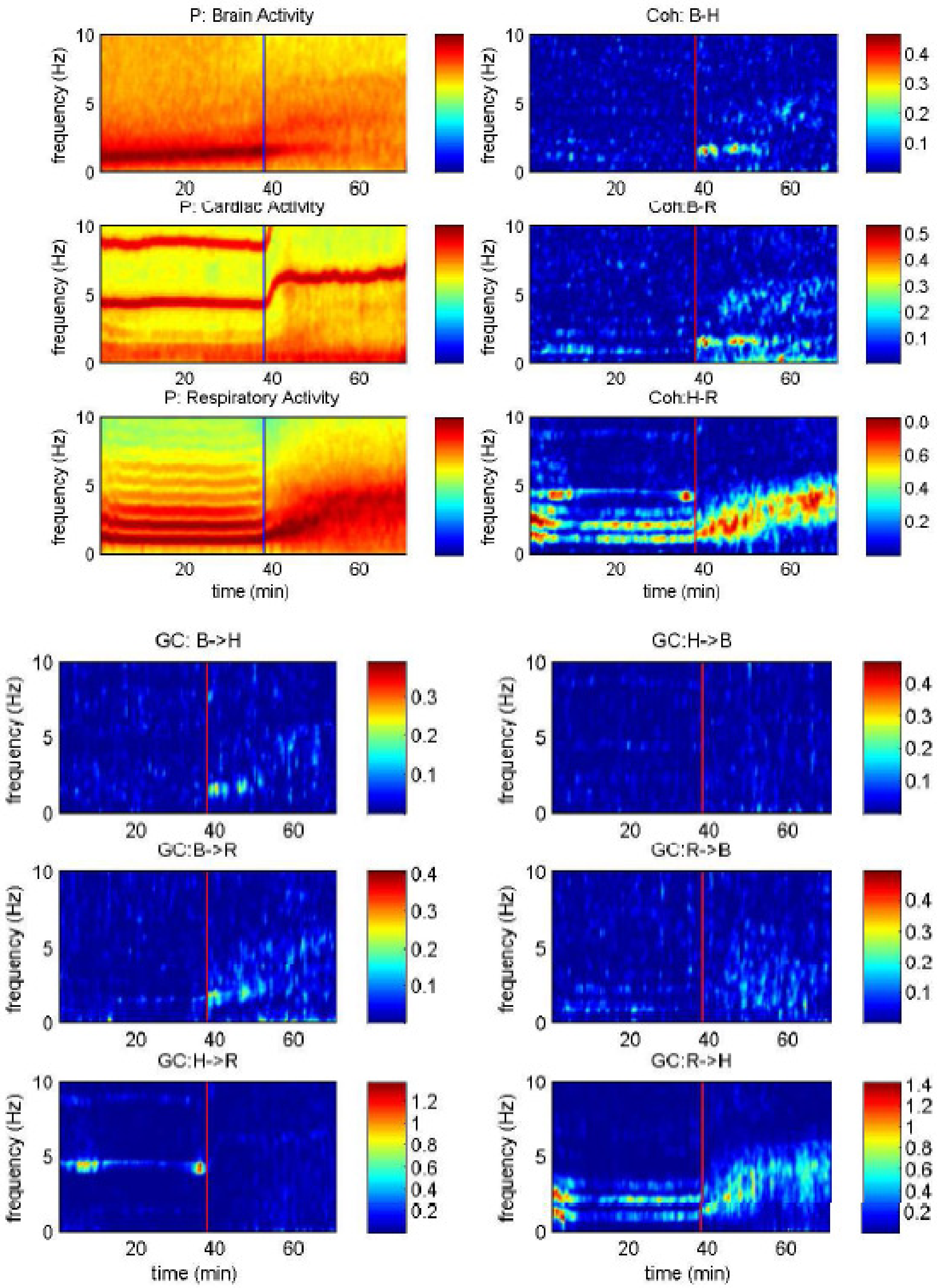

Fig S9. Rat 9 from KX group

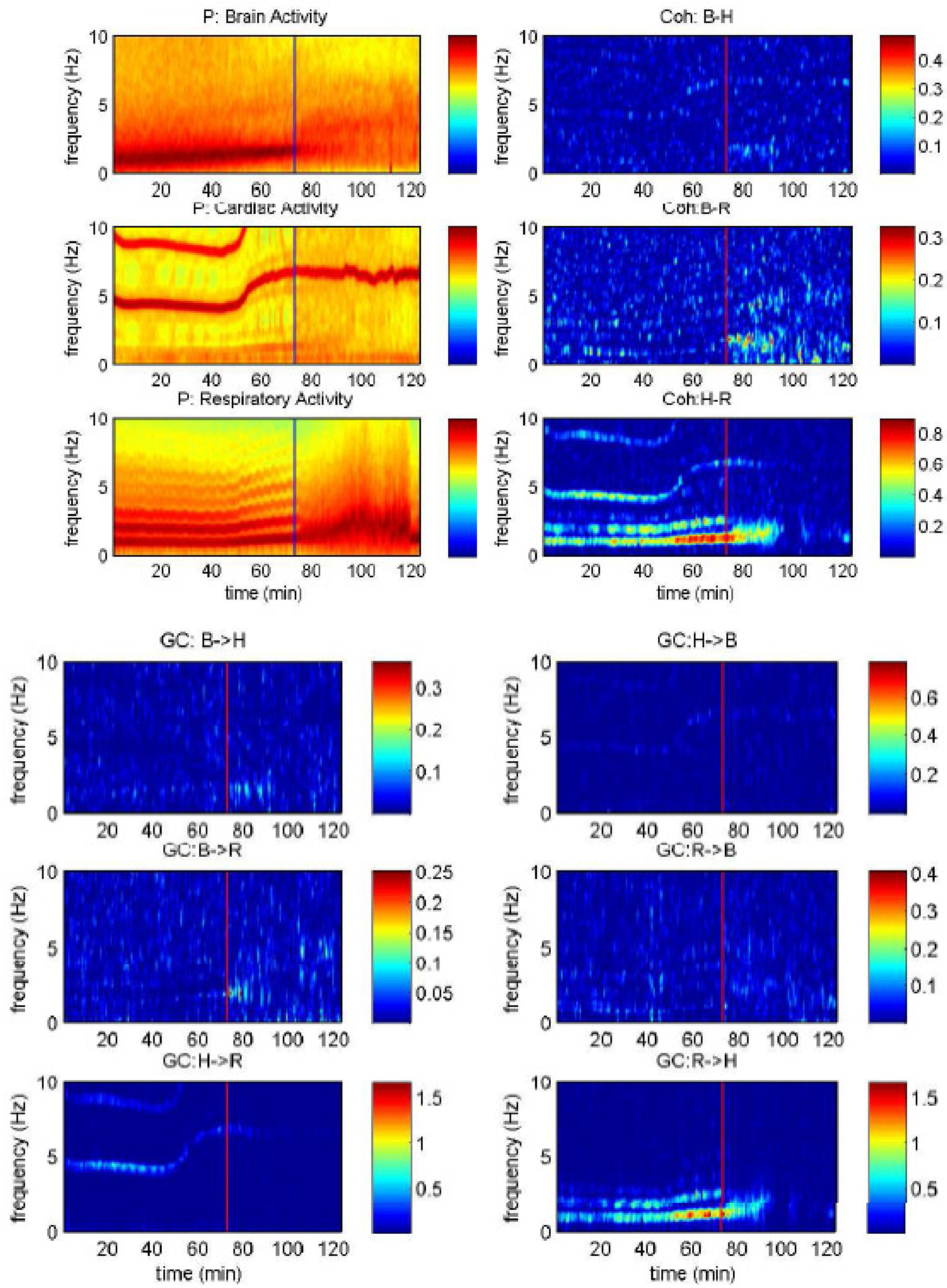

Fig S10. Rat 10 from KX group

(ii) PB group (Fig S11- S20):

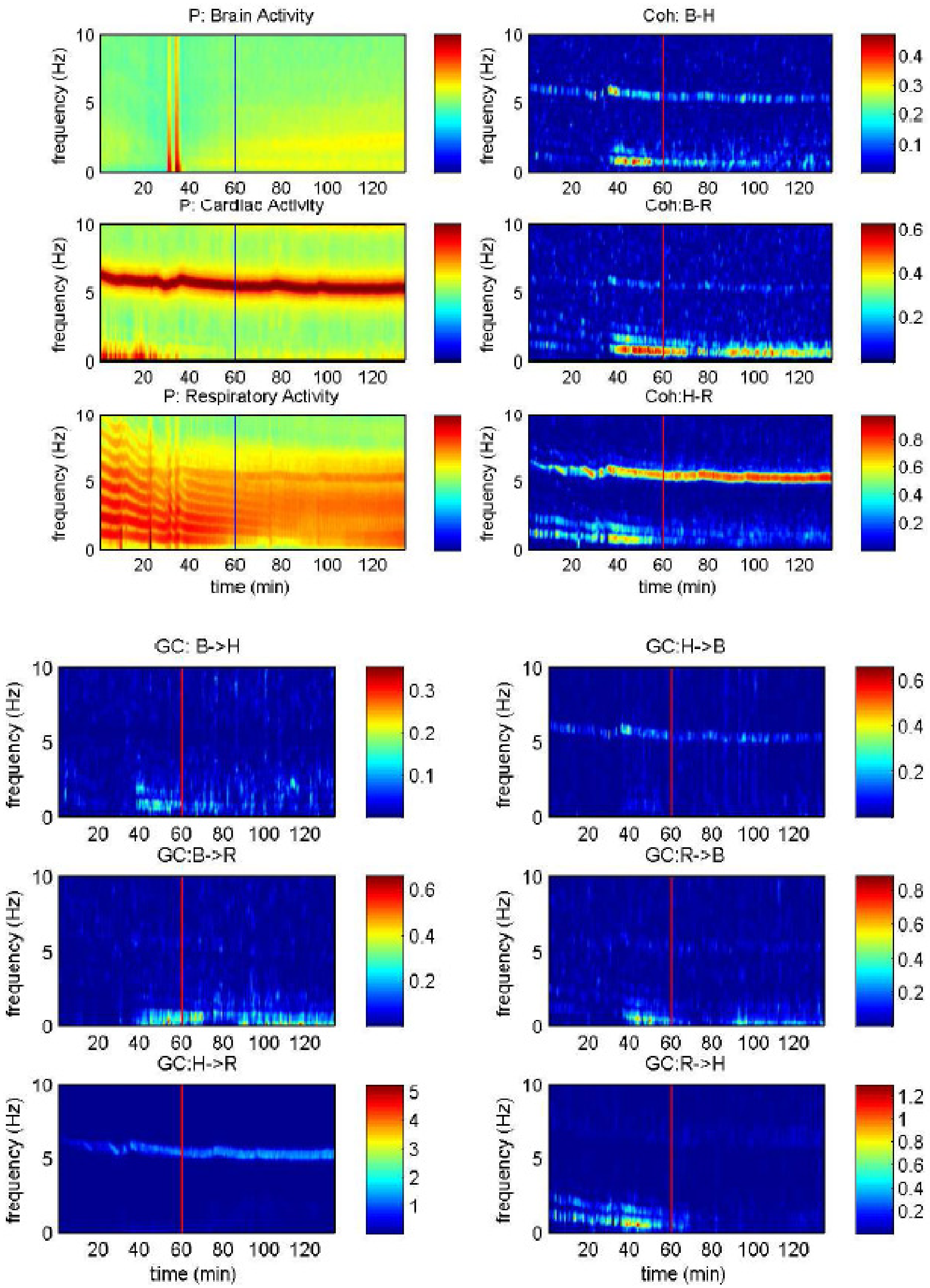

Fig S11. Rat 1 from PB group

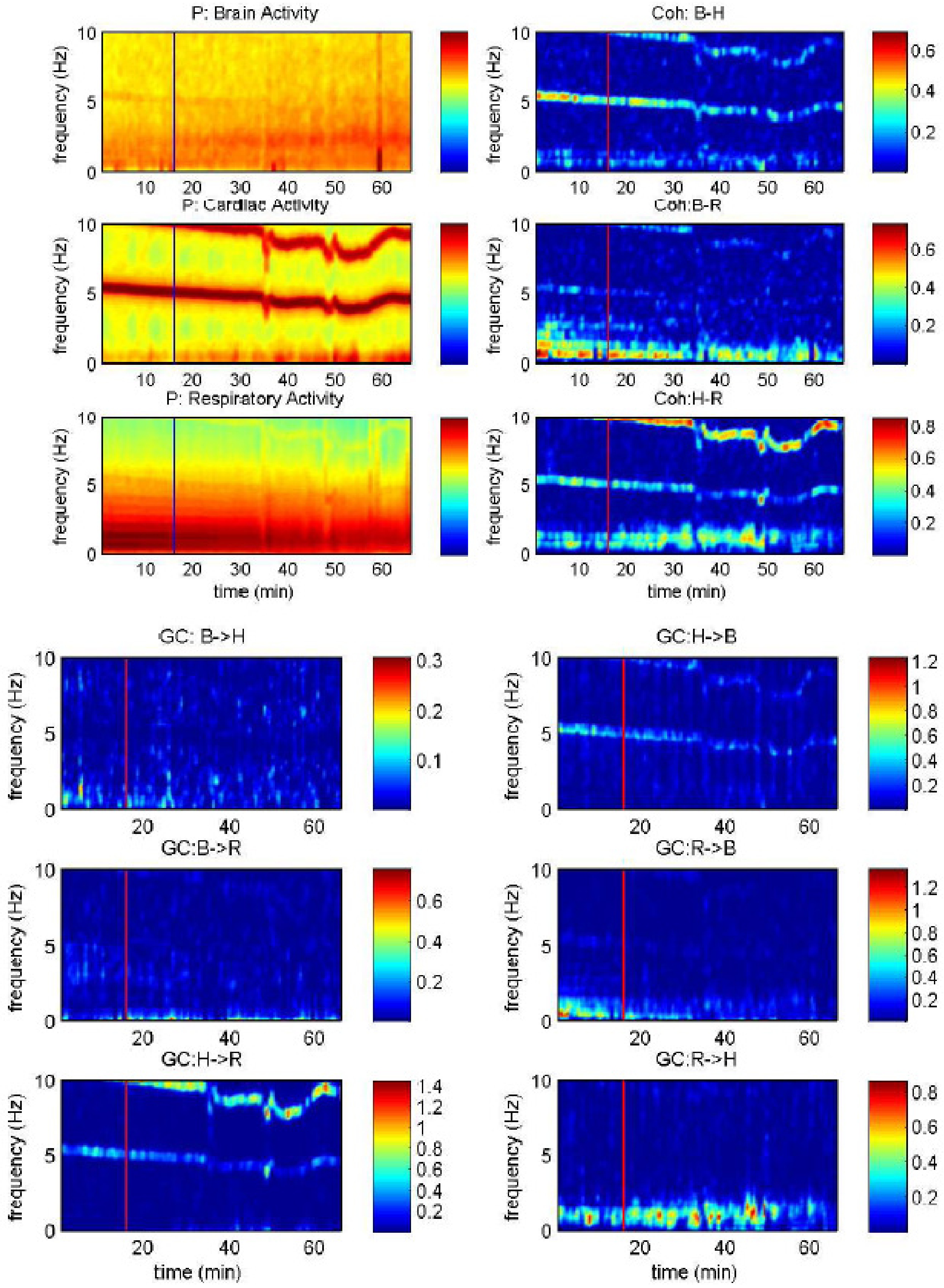

Fig S12. Rat 2 from PB group

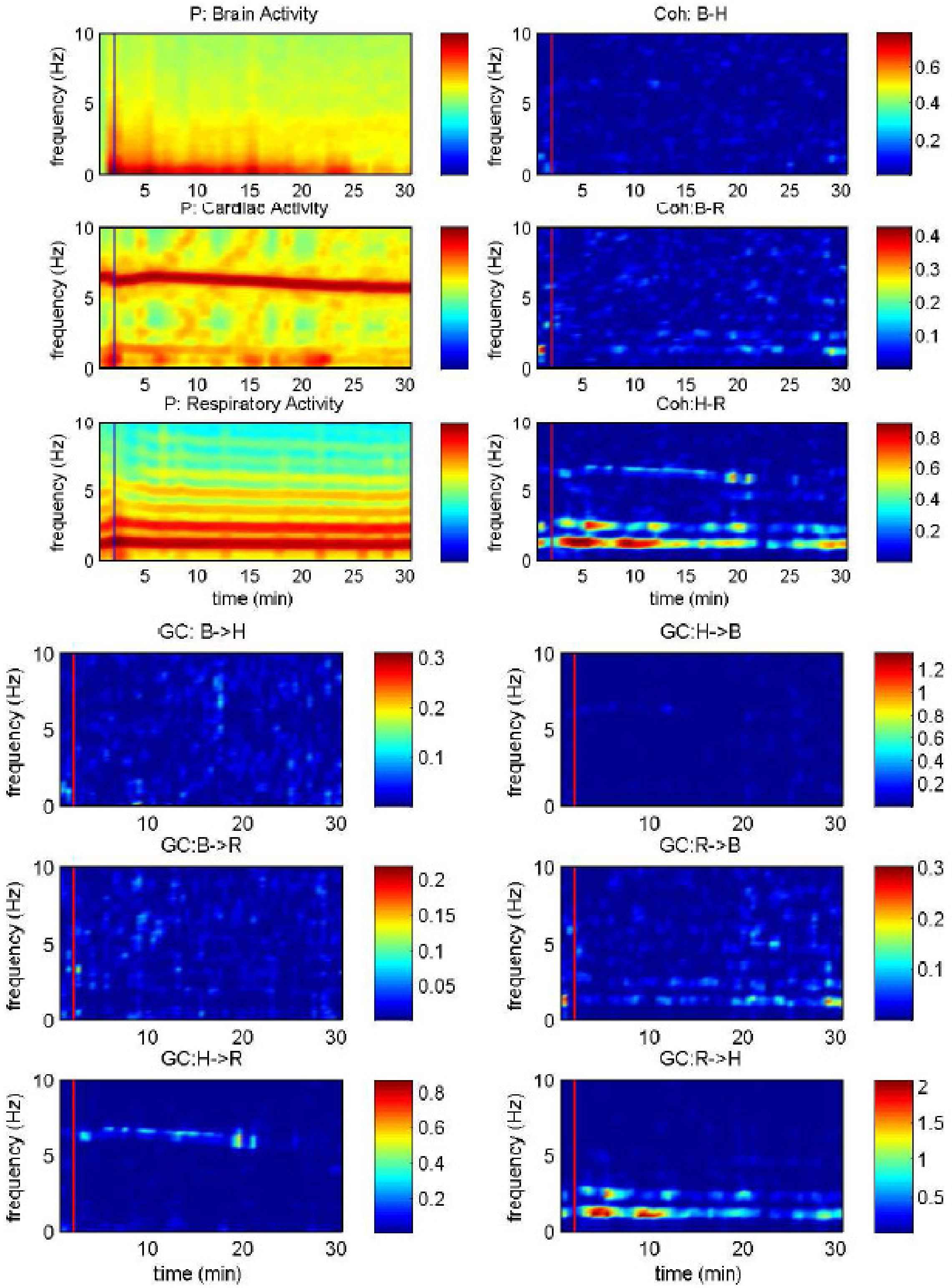

Fig S13. Rat 3 from PB group

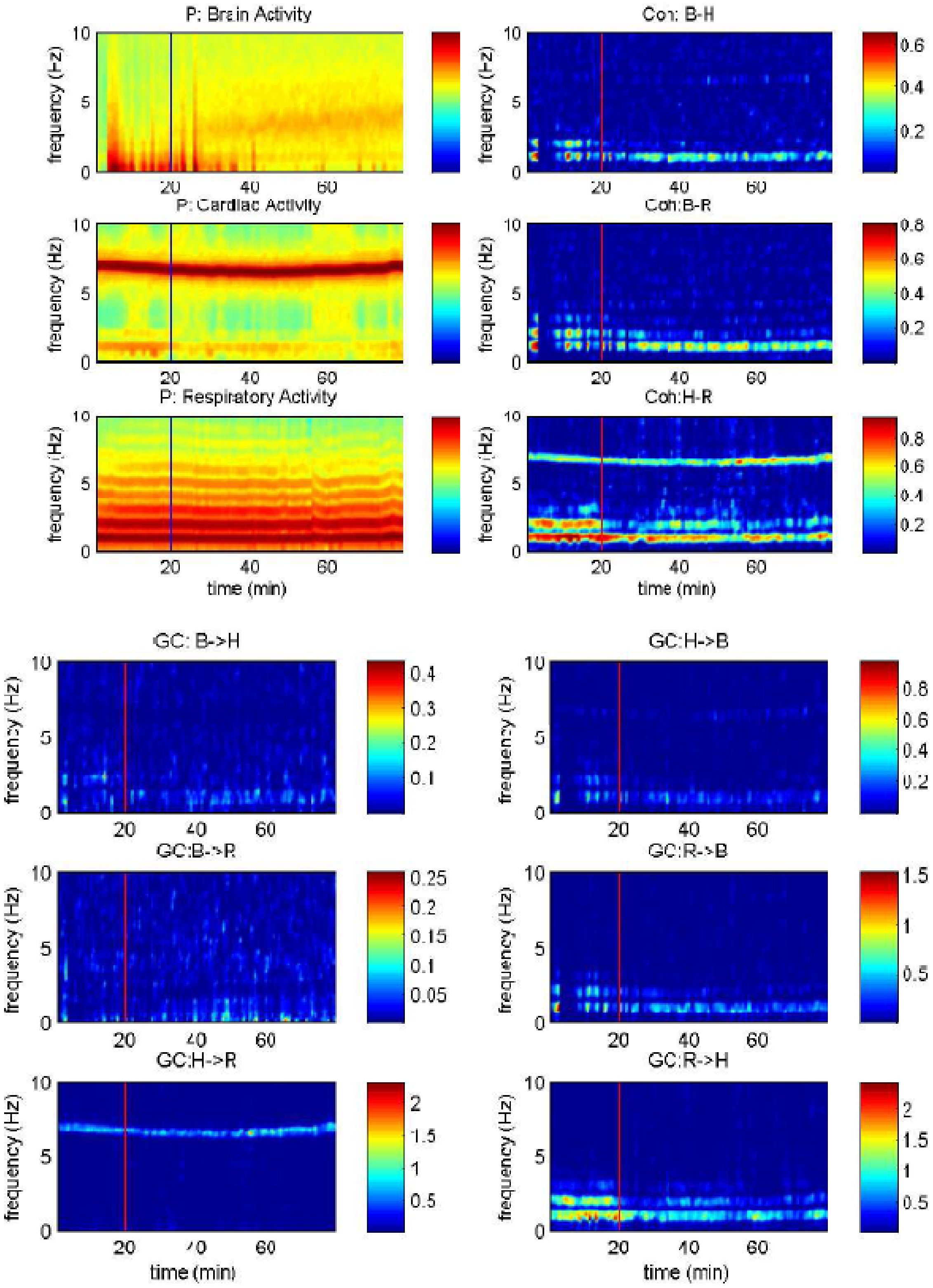

Fig S14. Rat 4 from PB group

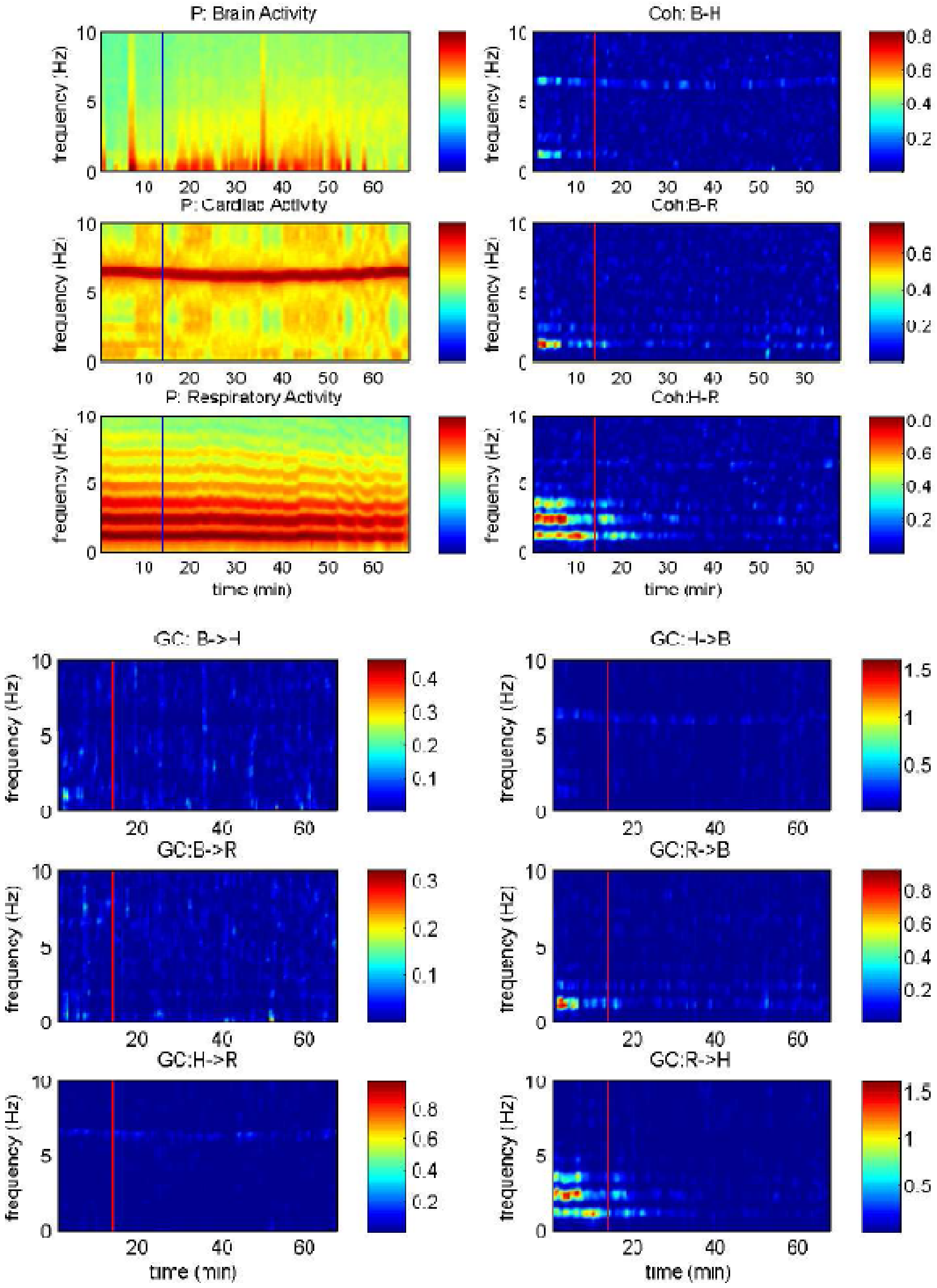

Fig S15. Rat 5 from PB group

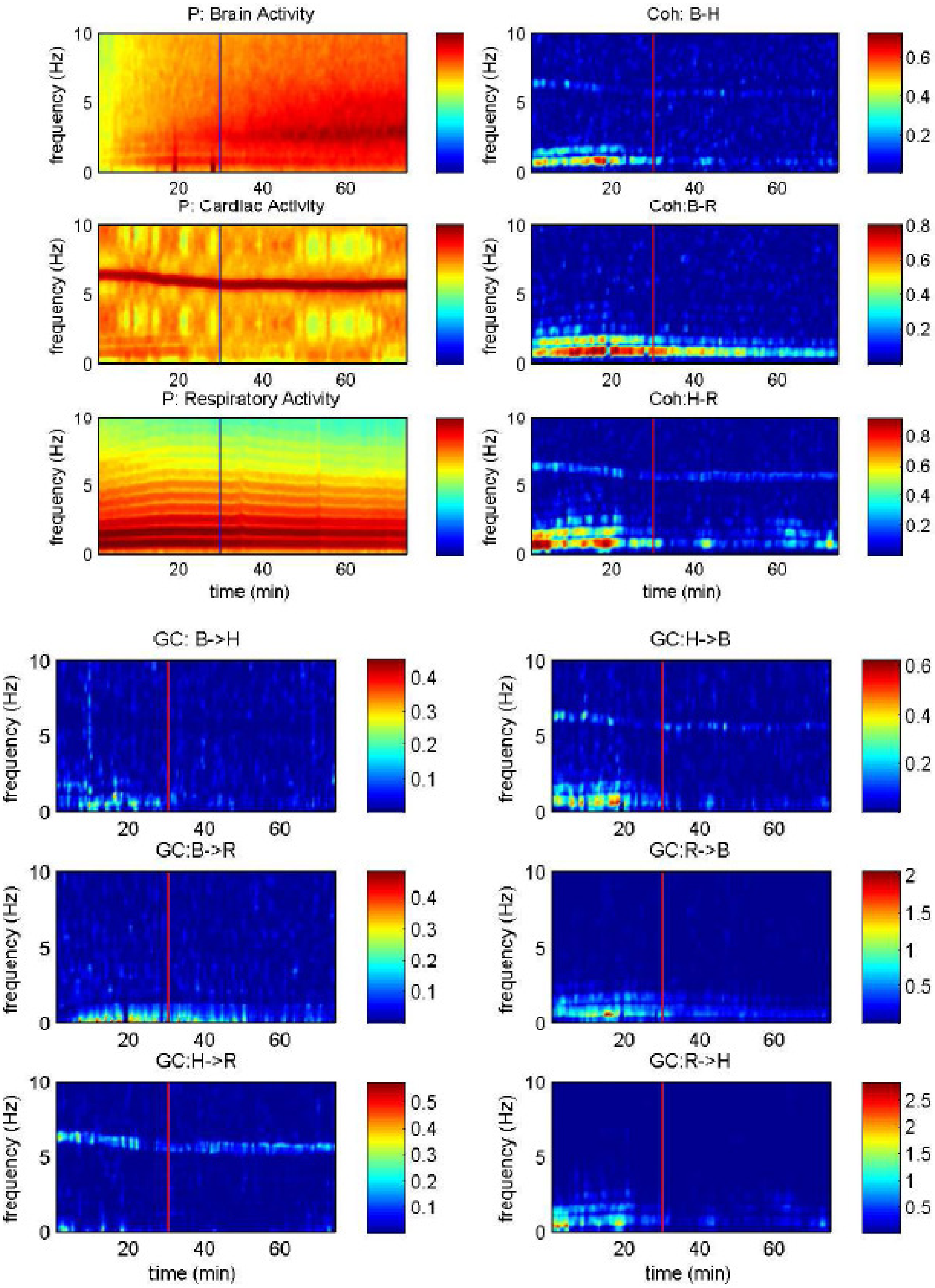

Fig S16. Rat 6 from PB group

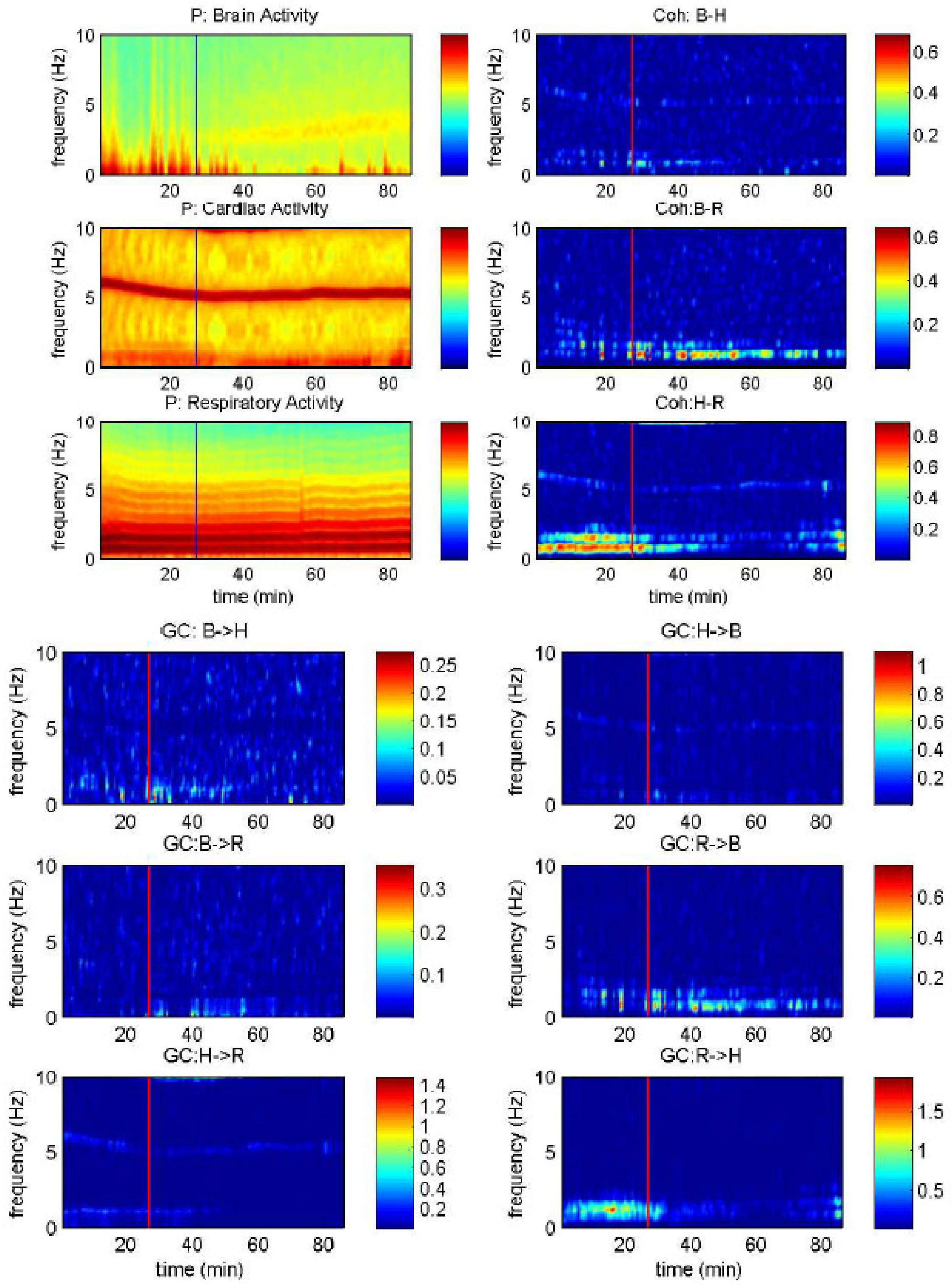

Fig S17. Rat 7 from PB group

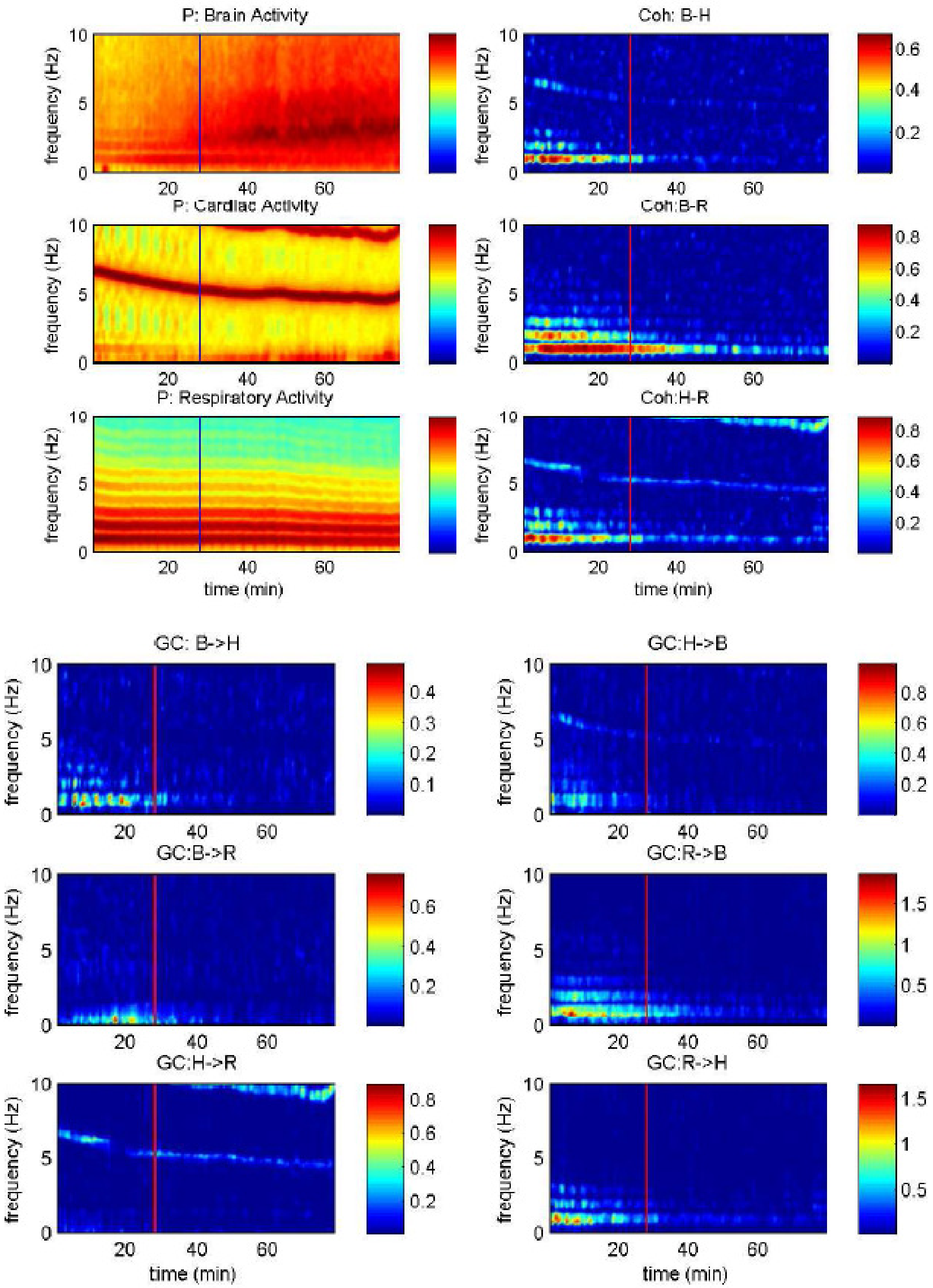

Fig S18. Rat 8 from PB group

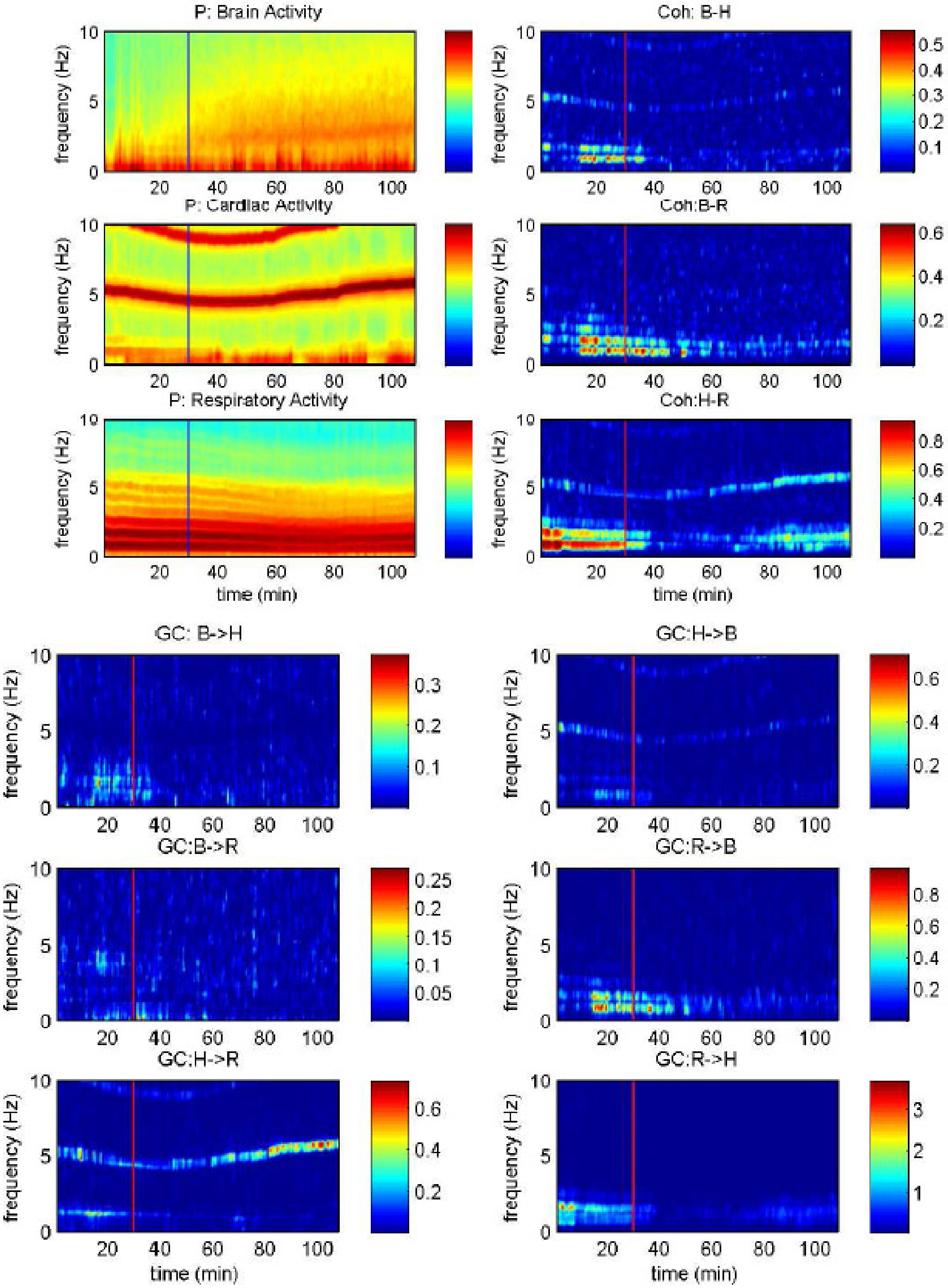

Fig S19. Rat 9 from PB group

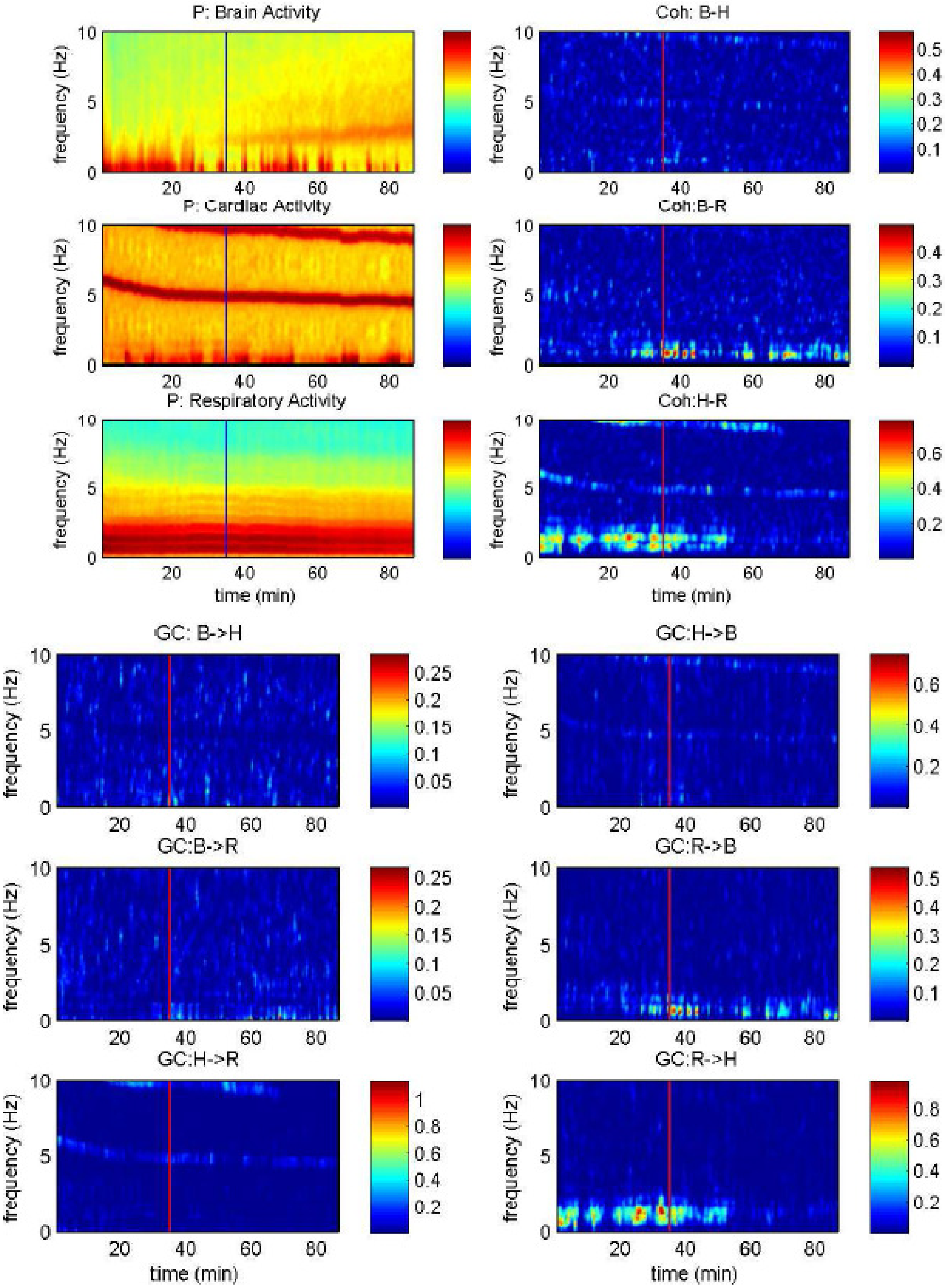

Fig S20. Rat 10 from PB group
